# How stressors disrupt honey bee biological traits and overwintering mechanisms

**DOI:** 10.1016/j.heliyon.2024.e34390

**Published:** 2024-07-10

**Authors:** Étienne Minaud, François Rebaudo, Padraig Davidson, Fani Hatjina, Andreas Hotho, Giulia Mainardi, Ingolf Steffan-Dewenter, Philippos Vardakas, Elise Verrier, Fabrice Requier

**Affiliations:** a Université Paris-Saclay, CNRS, IRD, UMR Évolution, Génomes, Comportement et Écologie, 91198, Gif-sur-Yvette, France; b Data Science Chair, Center for Artificial Intelligence and Data Science (CAIDAS), University of Würzburg, Würzburg, Germany; c Department of Apiculture, Institute of Animal Science, Hellenic Agricultural Organization 'DEMETER', 63200, Nea Moudania, Greece; d Department of Animal Ecology and Tropical Biology, Biocenter, University of Würzburg, Am Hubland, Würzburg, Germany

**Keywords:** Honey bee, *Apis mellifera*, Winter ecology, Winter mortality, Stressors, Feedback loop mechanism

## Abstract

High winter mortality of honey bees (*Apis mellifera*) has been observed in temperate regions over the past 30 years. Several biotic and abiotic stressors associated with winter colony losses have been identified, but the mechanisms and interactions underlying their effects remain unclear. We reviewed the effects of stressors on key overwintering biological traits, distinguishing between individual and colony traits. We found that disturbances at the level of individual traits can be amplified when transmitted to colony traits. By analyzing these cascading effects, we propose a concept of a feedback loop mechanism of winter mortality. We found that population size, social thermoregulation and honey reserve are integrative traits and can predict overwintering failure. Furthermore, we identified social thermoregulation as a good candidate for an early warning indicator. We therefore discuss existing tools for monitoring hive temperature to help mitigate the current high winter mortality of honey bees and support the sustainability of beekeeping.

## Highlights

• We reviewed potential mechanisms underlying the winter mortality of honey bee colonies.
• We propose a concept of a feedback loop mechanism of winter mortality involving key overwintering colony traits.
• Social thermoregulation fits as a candidate early warning indicator of colony mortality.
• Monitoring in-hive temperature would help to anticipate winter mortality of honey bee colonies.

## Graphical abstract

**Figure:**
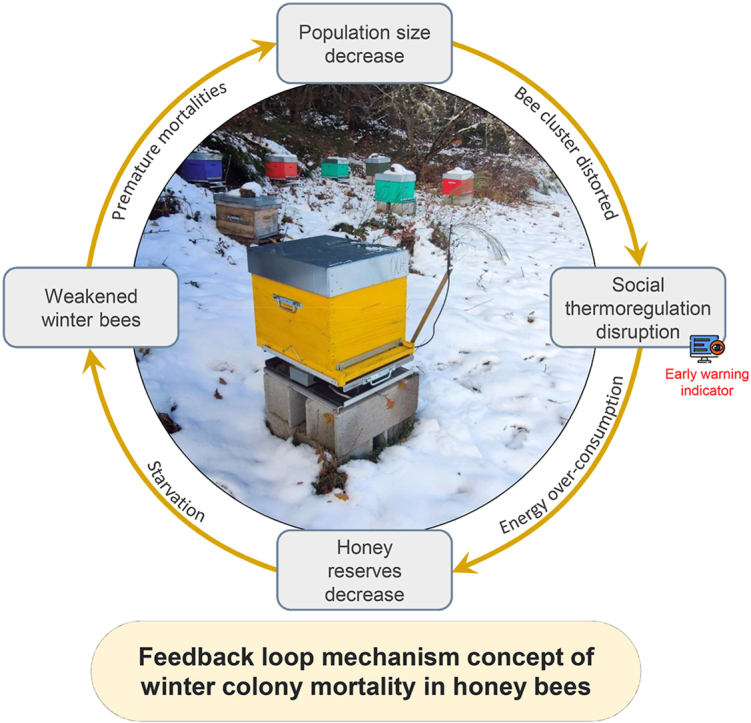

## 1 Introduction

Over the past 30 years, significant global decline in bees has been alarming both from the perspective of biodiversity conservation and on account of the essential pollination services that bees provide [[1], [2], [3]]. In particular, high winter mortality of the Western honey bee (*Apis mellifera*) has attracted attention due to its important role in human well being via crop pollination and beekeeping products. Economically harmful high winter mortality rates have been seen in several regions of the world, including the United States [4], Latin America [5], and Europe, where winter mortality rates can reach 25–50 % [6], potentially jeopardizing food security [7] and apiculture [8,9]. Various biotic and abiotic stressors that can weaken colonies alone or in combination have been identified [[10], [11], [12], [13], [14]]. As a social insect, the western honey bee is particularly sensitive to human-induced environmental change [15], relying on a fine-tuned colony organization and seasonal synchronization with abiotic conditions and floral resources. Loss of foraging habitat due to landscape conversion [16,17], climate change [[18], [19], [20]], and pesticide exposure [19,21] have been implicated in elevated winter mortality rates. Human activities have promoted the spread of biotic stressors (i.e., parasites, pathogens, and predators), particularly the ectoparasitic mite *Varroa destructor*, a main driver of colony loss [[21], [22], [23]]. This vector of several diseases has spread nearly worldwide after a host-jump from the Asian honey bee *Apis cerana* to the cosmopolitan western honey bee *Apis mellifera* [24]. Beekeeping practices are also involved (e.g., inbreeding by selection and introduction of non-native ecotypes [[25], [26], [27]], or the maintenance of high-density apiaries [28]), but good practices may limit losses [29]. However, despite a massive scientific mobilization to identify stressors, the ecological mechanisms underlying honey bee winter mortality are still poorly referenced.

In this paper, we aim to synthesize knowledge on the effects of stressors on the biological traits that underpin honey bee overwintering, both at the individual and colony level. Individual traits include morphology, physiology, immunity, performance and lifespan, while colony traits include population size, honey reserves, and social thermoregulation. In particular, we develop a conceptual framework for understanding how stressors occurring before and during autumn can deteriorate key overwintering traits through cascading effects. We explore the causal links between trait deterioration to provide clues to the mechanism of colony mortality. From this framework emerges a distinction between precursor and inclusive traits, which may enable the identification of traits that could serve as early warning indicators of winter colony failure. Additionally, we argue that further elucidation of the mechanisms underlying winter colony failure requires monitoring honey bees over the winter season. We conclude with a discussion of novel monitoring technologies that could shed light on how honey bee colonies survive –or fail to survive– the extreme life history hurdle of winter.

## 2 How honey bees overwinter?

### 2.1 Specific features of the winter bees

The western honey bee is an eusocial species living in a colony that survives from year to year through individual and social adaptations [30]. Colonies can survive for months in environments with extreme temperatures and without resources, such as dry seasons or temperate winters. These environmental constraints have physiological consequences, such as the interruption of brood rearing due to the lack of pollen intake. In the absence of brood, the survival of the colony is dependent on the last generations of workers that subsist on accumulated honey reserves until brood production can be resumed. In winter, this last generation of bees is called "winter bees", and their emergence in the autumn marks the beginning of the overwintering period. Winter bees differ from spring or summer bees in physiology, morphology, behavior, and longevity [[31], [32], [33]]. These differences begin to be noticeable in individuals emerging in the very late summer but become more pronounced in the emerging generations as winter approaches. The emergence transition from summer to winter bees is triggered by complex interactions between environmental factors, bee behavior, and physiology [31]. Decreased temperature, photoperiod, and floral resources lead to reduced foraging and nursing behaviors, triggering changes in pheromone levels (e.g., juvenile hormone or ethyloleate). In turn, these changes slow down maturation behavior, leading to the development of long-lived bees [31]. Indeed, winter bees live 130 days on average instead of 30 days for spring and summer workers [33,34]. This longevity is mainly due to their high vitellogenin levels, a glycoprotein known to increase lifespan by functioning in oxidative stress defense, immunity, behavioral control, and fat body development [35,36]. Compared to summer bees, winter bees present higher levels of hormones (except for hormones involved in foraging activity such as juvenile hormone) and higher levels of proteins [32]. Their immune system is enhanced by increased defense proteins (including vitellogenin), better hemocyte viability, and phagocytic response [37]. However, their nodulation and encapsulation responses are weaker than in summer bees [38]. They are also characterized by reduced flight and respiration capacities [37], and reduced activity of hypopharyngeal glands [32], known to be involved in nursing. As many other insect species, winter bees accumulate cryoprotective elements such as sugars and low molecular weight polyols during winter [39,40]. However, the individual cold resistance is relatively low compared to other Hymenoptera [41,42]. This low individual resistance is balanced by a social thermoregulation behavior [43].

### 2.2 Surviving the cold: social thermoregulation by the bee cluster

**Fig. 1:**
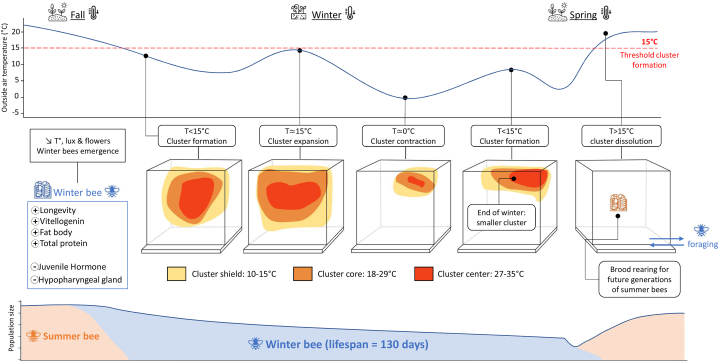
Overwintering of honey bees. Evolution of colony component individuals, population size [56], and cluster size [54] depending on time and external temperature. Winter bees (in blue) emerge at the end of autumn and survive until early spring to restart the colony. They differ from summer bees (in orange) in physiology, morphology, and lifespan (average 130 days against 30 days for summer bees). When the temperature drops below 18 °C, they perform social thermoregulation by forming a cluster in the hive. The mantle of the cluster (10–15 °C) is shown in yellow, the core (18–29 °C) in orange, and the center (27–35 °C) in red. The energy needed for this behavior is obtained by consuming the honey reserves made during the year. In spring, the surviving winter bees forage and rear the new summer bee brood. (

When the outside temperature drops below 15 °C, honey bees form a cluster and perform social thermoregulation to conserve energy and ensure survival [[44], [45], [46]] (Fig. 1). The temperature inside the hive is stabilize [47,48] with minimal heat production per individual but overall benefit from the heat produced by few individuals propagating to their neighbors [[47], [48], [49]]. Indeed, honey bees can temporarily reduce or generate heat by fanning or through micro-contractions of the thoracic muscles, called "shivering" [50]. Their thorax then can reach an average temperature of 30.4 °C (up to 36 °C) so that individuals are temporarily considered endothermic [48,49]. In the cluster, a group of central bees generate heat [49]. Their number gradually decreases towards the surface of the cluster, but their total number increases when the outside temperature decreases and vice versa [49,51] (e.g., 36 % at 14 °C, 15 % at 19 °C). The core of the cluster is maintained at an average temperature of 21.3 °C, the temperature being highest in the center (27–35 °C) and decreasing towards the surface [44,[52], [53], [54]] (18°C–29 °C). To limit heat loss, bees on the surface of the cluster form a mantle to reach 10–15 °C and never drop below 6.1 °C [44,52]. Mantle bees stick their hairy thoraxes together to form a shield [44]. They face the cluster center, positioning their head and thorax inward while exposing their abdomen to the outside [49]. Indeed, a counter-current heat exchange system allows honey bees to conserve heat in their head and thorax [50]. To save energy, winter bees take turns between phases of heat production, ventilation, or cluster shielding but rest most of the time [55].

The cluster limits the surface of exchange with the outside, decreasing it by more than 88 % compared to the total surface of all individuals [57]. Thus, the cluster shrinks in cold weather (e.g., at night) and relaxes in warmer conditions, allowing air to pass through to prevent overheating [44,49,58]. The cluster is usually ellipsoidal [44], and its size depends on colony strength and outside temperatures [44,47,54,58]. It does not contract further at an outside temperature of −5 °C but produces additional heat to survive [30]. The cluster is often located far from the nest entrances and away from the wind but moves, expands, and changes shape to keep access the honey reserves in the nest [44,54,58]. This ability to move reduces if the cluster is not able to generate enough heat, even during mild winter temperatures [44]. In laboratory, a cluster of bees can withstand temperatures as low as −80 °C for up to 1 h [43]. During the winter season, the colony can be considered a homeothermic superorganism [57] and can survive for months in extreme outdoor temperatures, as in Norway, where beekeeping was practiced up to 67° north latitude [59]. Within the bee cluster, other parameters are controlled, such as relative humidity (stabilized around 60 % [60]), or oxygen (stabilized around 15 % [61]). This low level of oxygen allows a decrease in the metabolic rate of honey bees and, therefore, a reduction of energy consumption [61] (i.e., the honey reserves).

### 2.3 Surviving the lack of food resource intake

During winter, floral resources are reduced, and climatic conditions limit foraging flights (e.g. cold temperatures). Thus, honey bee colonies usually stop food supply and feed on honey reserves stored during the summer when flower resources are abundant [30]. The availability of honey reserves and the ability of bees to consume it is, therefore, a critical criterion of winter survival [30,62]. To survive the winter and resume growth in spring, it is estimated that a colony must weigh at least 20 kg in autumn [63] (excluding the wooden materials of the hive) and include at least 4000 to 5000 bees [25,62]. These values vary depending on different factors such as genetic origin of the colony, overwintering location, hive type, and winter intensity [64,65], but the size of the population in autumn remains a good indicator of winter survival [25,63,66]. During winter, a colony loses on average 39 ± 13 g per day [65]. Smaller colonies exhibit lower rates of weight loss due to lower feeding rates [65] but also a higher average consumption per individual [67] and a higher individual mortality [68]. This could lead to a higher effort of individuals toward thermoregulation, which reduces the lifespan of winter bees [67].

Over the winter, the size of the winter bee population and the amount of honey reserves decrease and can sometimes lead to the mortality of the colony. The end of winter is a particularly sensitive period during which older winter bees must restart the colony. But adults may die before new brood can hatch and cause the population to rebound, leading to a collapse known as “spring dwindle” [56]. Winter mortality is therefore a natural phenomenon, but the current high winter mortality rates of managed colonies suggest that the overwintering mechanism has been disturbed by the emergence or accentuation of biotic and/or abiotic stressors [[10], [13]]

## 3 Biological traits involved and stressors affecting in the overwintering of honey bees

Stressors occur continuously throughout the year, but during the growing season, their effects can be masked by the strength of the colony and the availability of resources. Hypothesizing that stress factors have worsened the conditions for successful overwintering, several studies demonstrate that stressors occurring before and during autumn reduce winter survival [18,63,69,70]. Although these studies use correlative statistics between survival rate in spring and colony condition in autumn, less is known about the effects of stressors on overwintering biological traits, limiting the understanding of the mechanisms underlying colony mortality.

### 3.1 Key traits involved in the overwintering of honey bees

We synthesized current knowledge on the effects of stressors on key biological traits potentially involved in honey bee overwintering. For that, we performed an extensive literature search using the Web of Science (WOS) section of the bibliographical database ISI Web of Knowledge [71]. We used the topics function (TS) to define the search strings (i.e., the criteria key-words) in order to separate articles that had studied the topic of interest from those that only mentioned it, and excluded survey articles (i.e., studies quantifying winter colony losses using questionnaires) by filtering titles (TI). We included all literature from 1975 until November 2023. The complete search string was “TS= (honey bee* OR honeybee* OR Apis mellifera) AND TS= (winter* OR hibernation) AND TS= (loss* OR mortalit* OR collaps*) NOT TI= (survey)” (see **Supporting** **Information**). This systematic synthesis revealed 454 studies (1979–2023) and seven additional studies were considered from personal bibliographic databases. Applying four criteria: (1) original scientific article in English on honey bee biology, (2) study conducted during winter or on winter bees, (3) showing the significant effect of a stress factor (biotic or abiotic) on a biological trait (at individual or colony level), 74 studies were selected.

Among the selected studies, almost three quarters (73.0 %; 54 studies) show the disruptive impact of stressors on key overwintering traits (Table 1). The last quarter (27.0 %; 20 studies) investigated methods for mitigating these impacts. Key overwintering traits were identified at individual and colony levels. Individual traits were categorized into morphology (winter bee size, shape, and structure), physiology (levels of bioelements and related genes), immunity (pathogen prevalence, levels of immune and detoxification elements, oxidative stress), performance (movement abilities, senses, communication, or learning), and lifespan, covered in 27 studies (36.5 %). Colony traits were categorized into population size (adult population or brood size), honey reserves (reserve size or consumption), and social thermoregulation (cluster temperature, including its fluctuation), addressed in 23 studies (31.1 %). Both levels were studied in 24 studies (32.4 %). We find a good distribution of studies with regard to the trait level, but more disparity in the traits studied. Most studied traits included population size (36 studies, 48.6 %), bee lifespan (33 studies, 44.6 %), immunity (27 studies, 36.5 %), and physiology (21 studies, 28.4 %). Fewer studies dealt with honey reserves (17 studies, 23.0 %), and performance (14 studies, 18.9 %). The least studied traits are social thermoregulation (9 studies, 12.2 %) and morphology (7 studies, 9.5 %). Regarding stressors, over half of studies focused on almost one abiotic stressor (58.1 %; 43 studies), one-third on a biotic stressor (31.1 %; 23 studies), and the rest on combinations of both (10.8 %; 8 studies). Despite the consensus on the impact of combined effects [[10], [11], [12], [13], [14]], the number of stressor combinations that have been studied as a cause of weakened overwintering traits remains limited.

**Table 1:** Disruptive impacts of biotic and abiotic stressors on honey bee biological traits during winter at the individual or colony level. Vertical arrows indicate an effect toward the corresponding trait considered negative for colony survival (↓). Double horizontal arrows (↔) indicate a neutral or insignificant effect on the corresponding trait while at least one other trait is positively or negatively impacted. Empty cells indicate a lack of information.

		Individual traits					Colony traits			References
		Lifespan	Immunity	Physiology	Performance	Morphology	Population size	Honey reserve	Social thermo-regulation	
Abiotic stressors	Pesticide	↓↓↓ ↓↓ ↔↔	↓↓↓	↓↓↓ ↔	↓↓↓↓ ↓↓↓	↓↔	↓↓ ↔		↓↓↔	[72,[73], [74], [75],^76^,^77^,[78], [79], [80],^81^,^82^,^83^]
	Landscape composition^a^	↓↓↔	↓↓	↓↓↓	↓		↓↓ ↔	↓	↓↓	[[84], [85], [86], [87],^88^,^89^,^90^]
	Beekeeping practice		↓				↓	↓		[65,91]
	Climate	↓	↓	↓						[92]
Biotic Stressors	Parasite	↓↓↓ ↓↓↓	↓↓↓	↓↓↓	↓↓	↓↓	↓↓↓ ↓↓↓	↓↓	↓↓	[22,23,93,94,[95], [96], [97],[98], [99], [100], [101],^102^]
	Pathogen		↓↓	↓			↓			[38,103,104]
	Predator							↓		[105]
Combination of stressors	Parasite x pathogen	↓↓	↓↓↓	↓		↓	↓↓↓			[[106], [107], [108], [109], [110], [111]]
	Pesticide x pesticide	↓↓	↓↓	↓↓	↓			↓↔		[112,113]
	Parasite x landscape		↓	↓			↓	↓		[114]
	Pesticide x pathogen	↓			↓	↓		↓		[115]
	Parasite x beekeeping practices x climate	↓					↓			[116]
	Landscape x beekeeping practice						↓	↓		[25]
	Parasite x parasite							↓	↓	[117]
	Parasite x pesticide	↓					↓			[118]
	Pesticide x climate	↓								[119]
	Pathogen x pathogen	↓								[120]

a Include seven studies on resources availability and one study on the effect of electromagnetic fields.

We report disparities and gaps among the key overwintering traits studied (Table 1). These may be related to the importance attached to each trait by the scientific community, and to the technical capabilities to study it. At individual level, lifespan is important and can be evaluated relatively simply by survival experiments of cohorts of winter honey bees collected in autumn. Immunity, physiology, and morphology require bee sampling. The molecular analyses required for immunity and physiology can be performed on large samples and thus take much less time than the often-manual morphology measurements. Measuring honey bee performance is common in the study of pesticide effects, but we note that performance is rarely studied for other stressors. Interestingly, it is possible to artificially produce winter bees by simulating pre-winter conditions in the laboratory [121], which could facilitate the development of heavier monitoring experiments.

On the other hand, monitoring colonies during winter is challenging as opening the hive endangers the colony by disrupting social thermoregulation [122]. Population size and honey reserves can be estimated by visual inspection in early and late winter. Monitoring social thermoregulation requires electronic tools (e.g., temperature sensors), which may explain the low number of studies in this area.

### 3.2 Effects of abiotic stressors

Regarding the studies on abiotic stressors (51 studies in total), 35.3 % focus on landscape composition (18 studies). Most studies examine resource availability and/or quality, while one demonstrated electromagnetic fields disrupting the transition from summer to winter bees [84]. Studies found unfavorable effects from landscapes lacking semi-natural features [85] or offering poor pollen diversity [86]. In agricultural regions, colonies tend to have smaller adult populations, honey reserves, and unstable internal temperatures compared to those in semi-natural and mountainous areas [[72], [87], [114]]. Effects of nutritional stressors can be mitigated by artificial food supplementation during winter (e.g., sugar or pollen, with or without additives such as probiotics, vitamins, or herbs [41,66,[88], [123], [124], [125], [126], [127], [128], [129]]). The supplementation can improve winter bee weight [88], physiology [127,128], immunity [124,127], and longevity [127,128,88]. At colony scale, the supplementation before and during winter can support population size [66,123,124,129,130,131] and honey reserves [66,127,128]. On the other hand, supplementing too early (e.g., end of summer) can lead to excessive populations with over consumption of honey reserves before winter with potential risk to colony survival [66]. Nevertheless, positive effects of supplementation on individuals and colonies are non-systematic [132]. The excessive use of additives in artificial food supplementation can be toxic [88] or increase the prevalence of pathogens [125,126].

Pesticide was studied in one-third of studies (17 studies). One or more pesticides are tested in each study, and the predominant ones are neonicotinoid insecticides (e.g., imidacloprid or clothianidin, 11 studies), followed by herbicides (4 studies) and fungicides (3 studies). In winter, the hive can contain pesticide residues [133], which may expose winter bees to low doses for an extended period [112,113]. Pesticides can affect individual traits and prematurely age individuals by damaging mitochondria [73] or increasing oxidative stress [112,113] reducing lifespan [112,113,[74], [75], [76], [77], [78], [115], [118], [119]]. Nervous system may be impaired [113,74], which could explain disruption in learning capacities [75], communication abilities [75,79], movement [77], flight performance [72,79], or brood rearing [80]. In parallel with nervous system disturbances, over-consumption has been found [113], likely to compensate for this stress. Other colony-level impacts include social thermoregulation disruption [72,73], along with reduction in brood size, emergence rate, and adult winter bee population size [79,80]. The lack of knowledge concerning pesticide effects on winter bees (i.e., most studies on the effects of pesticides are carried out on summer bees) could lead to underestimate the real impacts of pesticide exposures on honey bees.

Beekeeping practices were considered in 19.6 % of studies (10 studies), including genetic selection and diversity [25,65,[91], [116], [134], [135], [136]], and hive type [130,135,137,138]. We note that only three studies considered the potential effect of subspecies on colony dynamics and survival [25,64,65]. They found that local subspecies had a stronger population [25] and, overall, a higher survival rate [64]. Moreover, some subspecies selected for production can consume more honey reserves in winter [65]. Two studies focused on the effect of intra-colony genetic diversity on the presence of parasites and pathogens but obtained contradictory results [91,136]. This could be explained by the presence of a resistant genetic lineage within low-diversity colonies, as tolerant strains exist and can be selected [116,134]. In summer, a greater genetic diversity of individuals in the colony promotes stable thermoregulation [139]. These results can probably be transposed to winter and impact social thermoregulation. Finally, a wooden hive rather than a polystyrene limits the reduction in population [130], and a cylindrical rather than a cubic hive limits reduction in population and reserves [137]. These differences may be explained by better heat conservation in these types of hives.

Only one study investigated climate effects on overwintering traits in real field conditions, revealing higher pathogen loads in warmer climates during winter [92]. We noted that another study showing that the appearance of brood at the end of winter is linked to temperature suggests that global warming could be desynchronizing colony and flowering phenology [140], which would be interesting to verify. The effects of climate can be artificially mitigated by placing hives indoors [134,[141], [142], [143], [144]]. Indoor overwintering can improve winter bee physiology [143,144] and population [134,141,144] and decrease consumption [142]. Indoor, limiting aeration may increase dioxide sufficiently to limit the prevalence of Varroa mites [135]. A heating device can also be installed and increases the population but also the number of Varroa mites [145].

### 3.3 Effects of biotic stressors

As expected, among studies involving biotic stressors (33 studies in total), the majority focused on the ectoparasite mite *Varroa destructor* (60.1 %; 20 studies). Varroa-infected honey bees in late autumn show divergent physiological and morphological traits compared to healthy winter bees, with less water content, fewer internal lipids and total protein, less vitellogenin, and more ecdysteroids [35,93,94]. Varroa mites are associated with higher pathogen loads [[106], [107], [108], [109], [110], [111]] through direct transmission as disease vectors [146,147] or through reduced immunity. Indeed, infested winter bees have a reduced number of immune cells [35,93], which could be linked to the reduction in vitellogenin. A reduction in lifespan has been demonstrated several times [22,93,106,109,95,96], which may partly explain the population declines observed during infestation [22,23,95,97]. Various treatments are available to control Varroa in order to limit losses [146,147]. Following the respective recommendations of each product (timing and frequency) is essential, otherwise produce efficiency can be reduced by more than 25 % and residues can accumulate in the hive and increase mite resistance [148]. For example, too high a dose of treatment (acaricide) can disrupt the physiology and reduce the immunity of winter bees [81].

The tracheal mite *Acarapis woodi* is another focus (12.1 %; 4 studies). This mite lives in the spiracles of honey bee, disrupting oxygen consumption, which impacts low-temperature flight ability and lifespan [98]. This disturbance also reduces heat production, impairing social thermoregulation and lowering the cluster's temperature and respiration [99]. Moreover, *A. woodi*-infected colonies exhibit lower pre-winter and post-winter populations and higher honey reserve consumption than healthy colonies [100,101]. We notice that only three parasites (*V. destructor, A. woodi,* and the small hive beetle *Aethina tumida*; see "combined effects”) have been studied for their impact on biological traits in the overwintering context, while colonies are attacked by more than eight major parasites overall [100,101]. For example, the impact of wax moths on overwintering is unknown (e.g., the wax moth *Galleria mellonella*). The presence of these lepidopterans in the hive could disrupt social thermoregulation by consuming combs.

Pathogens are the second most studied biotic stressor (39.4 %; 13 studies). This includes Varroa-transmissible pathogens such as Deformed Wing Virus (DWV) [38,106,109,110], Acute Bee Paralysis Virus (ABPV) [106,107,109,111], and potentially Chronic Bee Paralysis Virus (CBPV) [120], but also *Nosema ceranae* [115,106,[103], [104], [120]] transmitted between individuals. Thus, most of them are studied in combination with Varroa. Alone, DWV affects winter bee morphology and vitellogenin levels, reducing lifespan [115,106,109,111,120], and *N. ceranae* reduces physiologically essential bioelements [104]. At the colony level, all pathogens studied reduced population size through deaths of infected individuals and potentially leading to colony mortality [[106], [107], [108], [109], [110], [111]].

The Yellow-legged hornet *Vespa velutina* is one of the most significant predators of honey bees and has been considered in a single study [105]. In late autumn, hornets hovering at the hive entrance induce "foraging paralysis". Consequently, bees stay in the hive, thereby maintaining population size but depleting honey reserves just before winter.

### 3.4 Combined effects of stressors

Among the studies including combined effects (22 studies), 63.6 % focus on parasites (14 studies), in combination with pathogens [[106], [107], [108], [109], [110], [111]], parasites [117], pesticides [118], landscape composition [114], beekeeping practices [116], or climate [99]. We found two studies on pesticide-pesticide interaction [112,113], one on pesticide-pathogen interaction [115] and one on pathogen-pathogen interaction [120].

The combination of *V. destructor* and pathogens is the most studied [[106], [107], [108], [109], [110], [111]]. In this case, decreased immunity [110], increased infestations and infections [114,108,109], or decreased lifespan [106,109] have been reported at the individual level. At the colony level, population size is reduced [106,107,111]. The combined infestation of *V. destructor* and *A. tumida* disrupts cluster cohesion, resulting in a higher maximum core temperature and increased temperature fluctuations [117]. *V. destructor* has also been studied in combination with another parasite [117], beekeeping practices [116], landscape structure [114], and pesticides [118]. *V. destructor* infestation can obscure the beneficial effect of landscapes without intensive agriculture [114]. During *V. destructor* infestation and in cold environments, individuals selected for grooming have a decreased lifespan [116], probably due to an excessive metabolic cost of this behavior under unfavorable conditions. Finally, *V. destructor* infestation combined with neonicotinoids reduces lifespan and population size, effects that were not observed with simple exposure to these tested doses of insecticides [118].

Toxicity of pesticides can be exacerbated by pesticide mixtures [113]. Mixtures can disrupt the detoxification process, nervous system, defense against oxidative stress, metabolism, and immunity [112,113]. Surprisingly, toxicity of pesticide mixture can sometimes be higher at lower doses [112]. Decreasing of environmental temperature can also increase pesticide toxicity [119], which could create a loop with a social thermoregulation defect (triggered by pesticides or not). The combination of a herbicide and the pathogen *N. cerenae* causes swelling and tilting of the abdomen, altered movements, increased food consumption, and reduced lifespan [115].

## 4 Which mechanism involved?

### 4.1 Cascading effects in the disruption of overwintering traits

**Fig. 2:**
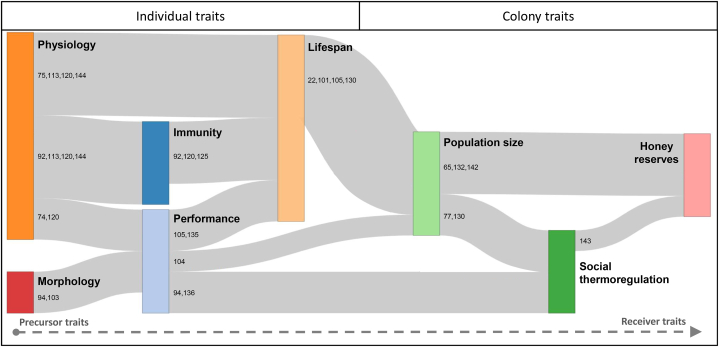
Causal links between key biological traits in honey bee colonies undergoing overwintering stress. The width of the links and the size of the biological traits are proportional to the number of studies concerned. We show horizontal arrows indicating the direction of reading of causal links using the package networkD3 in R [149]. The position of the traits is determined by calculating the number of causal effects of which it is the precursor relative to the number of causal effects it has received.

Interestingly, we found cascading effects among key traits involved in the overwintering of honey bees in 28.4 % of the reviewed papers (21 studies, Fig. 2). Disturbance of honey bee physiology can have detrimental effects of honey bee lifespan [85,92,93,102], performance [84,93], and immunity [112,92,93,102]. Bee immunity disturbance, in turn, shortens lifespan [112,93,109]. Disturbance of bee morphology also impacts individual performance [73,79], while disturbed bee performance leads to reduced bee lifespan [116,98]. Along this cascade of effects on individual traits, bee lifespan could be the most integrative trait, representing an accumulation of individual level effects (Fig. 2). However, the chain of causal links is not restricted to individual traits and can spill over onto colony traits. This is the case for disturbance of bee performance, impacting both population size [80] and social thermoregulation [73,99]; and bee lifespan impacting population size [22,118,116,95]. At the colony level, disturbance of population size can affect social thermoregulation [87,95] and honey reserves [66,97,105]. Energy loss due to the disruption of social thermoregulation affects honey reserves directly [117]. The effects of the colony's traits both converge on honey reserves, which could be the most integrative colony-level trait [11]. In these causal links, colony traits seem more integrative than individual traits, as they accumulate disturbances from each other and all the individual traits. Thus, we identify population size, social thermoregulation, and honey reserves as better predictors of overwintering failure. They are good candidates as early warning indicators of winter mortality as long as accurate monitoring tools are available.

The causal links found between all traits for the most studied stressors (i.e. *V. destructor* and pesticides) give insights into potential mechanisms leading to colony mortality. For instance, adult winter bees exposed to pesticides (neonicotinoids, fungicides, or herbicides) show reduced immunity [113,82] and thus increased parasite and pathogen levels such as the Varroa mite [83]. The Varroa-infested colony begins overwintering with reduced honey reserves and population size [97]. This population includes infested winter bees that are morphologically, physiologically, and immunologically weakened [35,93], leading to a decrease in their lifespan and an increase in their probability of dying before the end of winter [22,93,106,109,95,96,102]. In addition, pesticides can increase the level of damaged mitochondrial DNA in the thorax of exposed honey bees, which can also represent accelerated aging and could lead to a drop in energy production and, thus, a failure of thermoregulation [73]. Coupled with the small population, thermoregulation is disturbed, and the internal temperature is lower and/or less stable [72]. The lower environmental temperature increases pesticide toxicity [119] but also metabolic rate of behaviors, such as grooming [116]. This may explain why colonies with low populations show higher consumption per individual [67,150] and higher individual mortality [68]. Over winter the colony weakens and if the population falls below a critical threshold, social thermoregulation may fail due to an insufficient number of winter bees in the colony [95].

These more studied stressors show cascading effects between traits, which can give a more global view of the mortality mechanism. The accumulation of disturbances on an individual scale impacts the colony. As the colony weakens, the impact of stressors can become increasingly significant. Overwintering failure induced by *V. destructor* can, therefore be facilitated by exposure to neonicotinoids [118], the presence of another parasites [117], or diseases [[106], [107], [108], [109], [110], [111]]. In addition to their own effect, stressors increase sensitivity to other stressors by combined effects [10].

### 4.2 Potential feedback loop underlying winter mortality

**Fig. 3:**
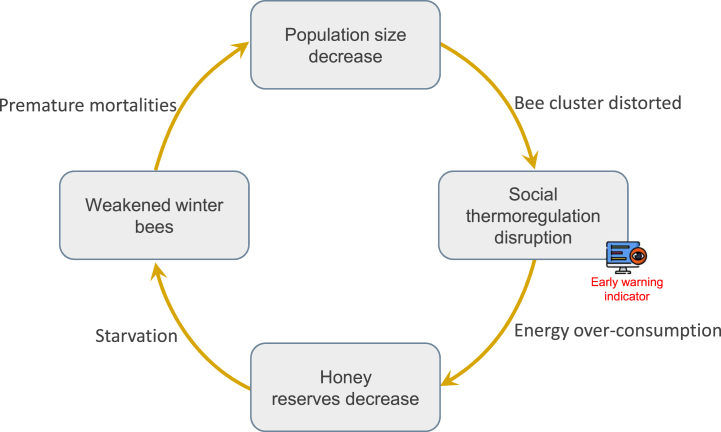
Concept of the feedback loop mechanism of winter colony mortality in honey bees. The premature mortality of winter bees reduces the population size, negatively affecting the size and cohesion of the cluster. The distorted bee cluster (e.g., a misshaped cluster and/or loss of heat per bee) affects social thermoregulation, resulting in over-consumption of honey reserves. Starvation due to decreased honey reserves weakens winter bees. Social thermoregulation is a good candidate as early warning indicator of winter colony mortality, as it is an integrative trait that can be accurately monitored by in-hive temperature sensors.

This first overview provides clues to both mechanisms of overwinter and winter mortality. To survive the winter, the social thermoregulation of honey bees is optimized to produce sufficient heat while consuming a minimum of energy (i.e., honey reserves). Any deviation from this optimum results in higher energy expenditure, which in turn leads to over-consumption and weakens individuals. This optimum is favored by a large population but individuals may die over winter (if their longevity is insufficient, due to infection or infestation, or premature aging caused by excessive energy expenditure). Thus, we suggest a feedback loop mechanism of winter colony mortality involving key overwintering traits (Fig. 3). The premature mortality of winter bees could reduce the size of the population, leading to distorted bee cluster and then impacting social thermoregulation. Fewer individuals would provide a misshaped cluster and/or loss of heat per bee. This social thermoregulation disruption leads either to an over-expenditure of energy per bee to maintain the optimum temperature, or to difficulties in reaching and maintaining the thermal optimum. In both cases, this results in higher energy costs and over-consumption of reserves. The weakening of individuals by starvation can lead to the death of new individuals, further reducing the population size. The colony will collapse, either for lack of individuals to produce and maintain a minimum temperature, or for lack of honey reserves. We suggest this concept of feedback loop mechanism of colony mortality, although mobilization is needed to study the links involved and the potential disturbances from all stressors that can take part in the mechanism. The energy overhead of disrupted social thermoregulation could also premature ageing of individuals and accelerate this feedback loop. It would be interesting to explore this hypothesis, for example, by monitoring survival of individuals in clusters of different sizes.

## 5 Monitoring key traits to anticipate winter mortality

We suggest that the winter mortality of honey bees may result from a feedback loop mechanism involving key colony traits (i.e., population size, social thermoregulation, and honey reserves). Thus, monitoring colony traits over the winter could enable the detection of early warning indicators of ongoing collapse mechanism, which can help beekeepers anticipate colony mortality and support decision-making. Early warning indicators of winter mortality have already been identified at the level of individual traits with vitellogenin level or by assessing pathogen loads [151]. However, these measurements require pre-winter sampling and analysis, which can be invasive (e.g., opening the hive during winter), time-consuming and expensive, challenging their routine uses by beekeepers. But the expansion of precision beekeeping, defined as using technology to monitor colonies to minimize mortality risks and maximize production [152,153], seems to be a promising solution. The advancements in information and communication technologies (ICT), including miniaturization, cost reduction, and diversification, have expanded their usage in beekeeping. Current uses of ICT mainly focus on monitoring swarming and honey production from spring to autumn. Still, they could be extended to monitor metrics indicative of key colony traits during winter.

Colony weight is a candidate measurement that can be controlled using connected scales, the most common tool in precision beekeeping and readily available on the market. The monitoring is mostly autonomous and is often connected to a network to automatically send data to a server. However, overwintering weight control makes it challenging to estimate key colony traits directly, as it is impossible to accurately disentangle the effects of population size and honey reserves on weight dynamics.

We therefore suggest that monitoring the in-hive temperature [154] to assess social thermoregulation is a relevant candidate early warning indicator of colony health and winter mortality. A rapid drop of in-hive temperatures can be a sign of ongoing collapse [52]. Lower average in-hive temperature over winter may be a sign of stressed colonies [87] anticipating collapse, as well as observing important fluctuations in internal temperatures. Fluctuating in-hive temperature can indicate difficulties in adjusting social thermoregulation to external temperatures [87,72,117].

Many temperature-monitoring tools are available to monitor social thermoregulation, with advantages and disadvantages (Table 2). Thermal cameras have been used to measure in-hive temperature with the advantage of not disturbing the colony. However, the thickness of the hive prevents an accurate measurement of the temperature and, thus, the detection of the cluster. Therefore, thermal imaging can determine whether a colony is alive or dead but it cannot provide accurate estimates of bee cluster location and area [155]. Temperature sensors in the hive are more precise and commonly used in precision beekeeping for colony monitoring [156], especially during the growing season (spring to autumn), to help detect brood rearing (i.e., in-hive temperature regulated around 35.5 °C). They are less used in winter, as interpretation is not as straightforward due to the dynamic nature of the cluster. Temperature sensors can range from one sensor inside the hive [72,157] to more than 60 sensors in a frame [52,158], substantially increasing the accuracy of the spatialization, position, size, and shape of the bee cluster. These tools have been used mainly under laboratory conditions and could be improved by developing a fully (e.g., with solar panels) or semi-autonomous system (e.g., with large capacity batteries [159]). With future work, they could enable to plan support or rescue interventions targeted only at weak colonies. A recent study proposes to install a robotic frame that monitors temperature and can produce heat either continuously to save the cluster energy, or curatively if it falls below the critical 10 °C threshold [52]. With less resource, colonies detected as weak can be supplemented, but those detected as dying require more substantial intervention such as indoor transfer, heating device or even a merging of several weak colonies to try and save at least one.

**Table 2:** Advantages and disadvantages of existing tools for measuring the temperature in the hive to monitor thermoregulation and clustering in winter. Tick (✓) & cross (✗) indicate whether the tool is adapted/not adapted for this purpose. Tools to detect the cluster allow for knowing its location, size, or shape. We consider a tool to be carrying out long-term continuous measurements if it operates over the winter (4/5 months) without intervention. We set an affordable threshold at 200 euros per colony.

Tools	Affordable price	Accurate measures	Continuous long-term measurements	Battery powerable	Cluster detection	References
Mobil wired sensor networks	✓	✓	✓	✓	✓	[158,160,161]
non-mobile wired sensor networks	✓	✓	✓	✗	✓	[52,54,162]
Multiple stand-alone loggers	✗	✓	✓	✓	✓	[87]
Stand-alone logger	✓	✓	✓	✓	✗	[72,83]
Smartphone thermal camera	✓	✗	✗	✗	✗	[163]
Thermal camera	✗	✗	✗	✗	✗	[155]

## 6 Concluding remarks

Overwintering of honey bees is achieved through individual fitness traits and colony performance. Many known stressors can affect these biological traits, but understanding their effects on overwintering is necessary to understand the mechanism of winter mortality. Here, we show that the effects of stressors on overwintering mechanism of honey bees are not well understood and that studying biological traits over the winter is challenging. This lack of information limits our overall understanding of winter mortality, and its potential dependence of different colony life histories and subspecies. We report disparities in research efforts both for stressors and traits studied. The disproportionate knowledge of *V. destructor* and the effects of pesticides may reflect a greater appreciation of their role as overwintering stressors. Moreover, the effects of some stress factors, such as climate change, remain unclear due to limited research [164], challenging the predictions of winter losses according to climate change projections. In addition, although combined effects of stressors are often invoked to explain bee decline [10,13], much less is known about the combined effects of stressors on honey bee overwintering. Furthermore, colony traits are less studied than individual traits, since conventional monitoring is impossible during the winter period, turning the hive into a black box.

As identifying causal links between traits provides clues to the mechanisms of mortality, we suggest that population size, social thermoregulation and honey reserves are integrative traits that accumulate disturbances from individual traits and are thus better predictors of overwintering failure. Among these predictive traits, we identified social thermoregulation as a good candidate early warning indicator. Moreover, we suggest the concept of a feedback loop mechanism of colony mortality for which the overwintering mechanism is centered on the colony's ability to optimize its social thermoregulation to limit energy loss and maintain its population until spring. Directly or indirectly, stress factors can disrupt this mechanism, driving the colony into a feedback loop of deterioration until a critical threshold is reached, triggering mortality [11]. Identifying these thresholds could allow the establishment of monitoring systems to anticipate colony mortality, thereby helping to reverse the current global trend of colony losses and support the sustainability of beekeeping.

## Funding

This work has received funding from the European Union's Horizon 2020 research and innovation program under grand agreement no 862665 ICT-AGRI-FOOD and with the funding organisations: Agence Nationale de la Recherche in France (ANR; ANR-21-ICAF-0001), Bundesanstalt für Landwirtschaft und Ernährung in Germany (BLE; FKZ 2820ERA17H), and General Secretariat for Research and Innovation in Greece (GSRI; Τ12ΕΡΑ5-00098/40546).

## Disclosure statement

The authors reported no potential conflict of interest.

## Data availability statement

The data presented in this manuscript are available in the supplemental information.

## CRediT authorship contribution statement

**Etienne Minaud:** Writing – review & editing, Writing – original draft, Visualization, Methodology, Investigation, Formal analysis, Data curation, Conceptualization. **François Rebaudo:** Writing – review & editing, Writing – original draft, Supervision, Methodology, Conceptualization. **Padraig Davidson:** Writing – review & editing, Visualization. **Fani Hatjina:** Writing – review & editing. **Andreas Hotho:** Writing – review & editing. **Guilia Mainardi:** Writing – review & editing. **Ingolf Steffan-Dewenter:** Writing – review & editing. **Philippos Vardakas:** Writing – review & editing. **Elise Verrier:** Writing – review & editing. **Fabrice Requier:** Writing – review & editing, Writing – original draft, Validation, Supervision, Resources, Project administration, Methodology, Funding acquisition, Formal analysis, Conceptualization.

## Declaration of competing interest

The authors declare that they have no known competing financial interests or personal relationships that could have appeared to influence the work reported in this paper.
