# Supplementary material for: How stressors disrupt honey bee biological traits and overwintering mechanisms
Source: Heliyon. 2024 Jul 10;10(14):e34390. doi: 10.1016/j.heliyon.2024.e34390 (PMC11301357; doi:10.1016/j.heliyon.2024.e34390)
Supplement: Multimedia component 1 [file mmc1.docx]

**Supplemental Information for:**

**How stressors disrupt honey bee biological traits and overwintering mechanisms**

**Appendix 1: Detailed method of the article selection**

We performed an extensive literature search using the Web of Science (WOS) section of the bibliographical database ISI Web of Knowledge (Clarivate, A.P., and Thamson Scientific Web of Knowledge. Web Knowl. http://apps.webofknowledge.com/). We used the TI (Tittle) and TS (Topic) functions to define the search strings (i.e. the criteria key-words) in order to separate articles that had studied the topic of interest from those that only mentioned it. We included all literature from 1975 to November 2023 mentioning honey bees and overwintering and mortality. To refine the search, we excluded articles whose title mentioned "survey" in the search string (i.e. NOT TI=(survey)). Using the WOS "refine results" index, we discarded articles in a language other than English (15 articles) and documents other than original articles such as review articles, proceeding papers, book chapters and editorial material (27 articles). The complete search string was TS=(honey bee* OR honeybee* OR Apis mellifera) AND TS=(winter* OR overwinter* OR hibernation) AND TS=(loss* OR mortalit* OR collaps*) NOT TI=(survey). This search string can be found with the following link:

https://www.webofscience.com/wos/woscc/summary/6cd851ab-82a8-4269-9892-499dc71a3626-b2e52a0d/relevance/1.

This systematic synthesis revealed 454 studies. The titles and abstracts, and if necessary the full texts of the articles, were read for filtering using three criteria: (1) original scientific article in English on bee biology, (2) study carried out in winter and/or on winter bees, (3) showing the significant effects of a stress factor (biotic or environmental) on a biological trait (at individual or colony level). Articles have been discarded whenever one of the criteria was not achieved, leading to a set of 67 articles (**Figure A**). Seven additional studies were considered from personal bibliographic databases for a final corpus of 72 studies selected (**Figure S1**). The full reference of all articles is available in the **Table A**.


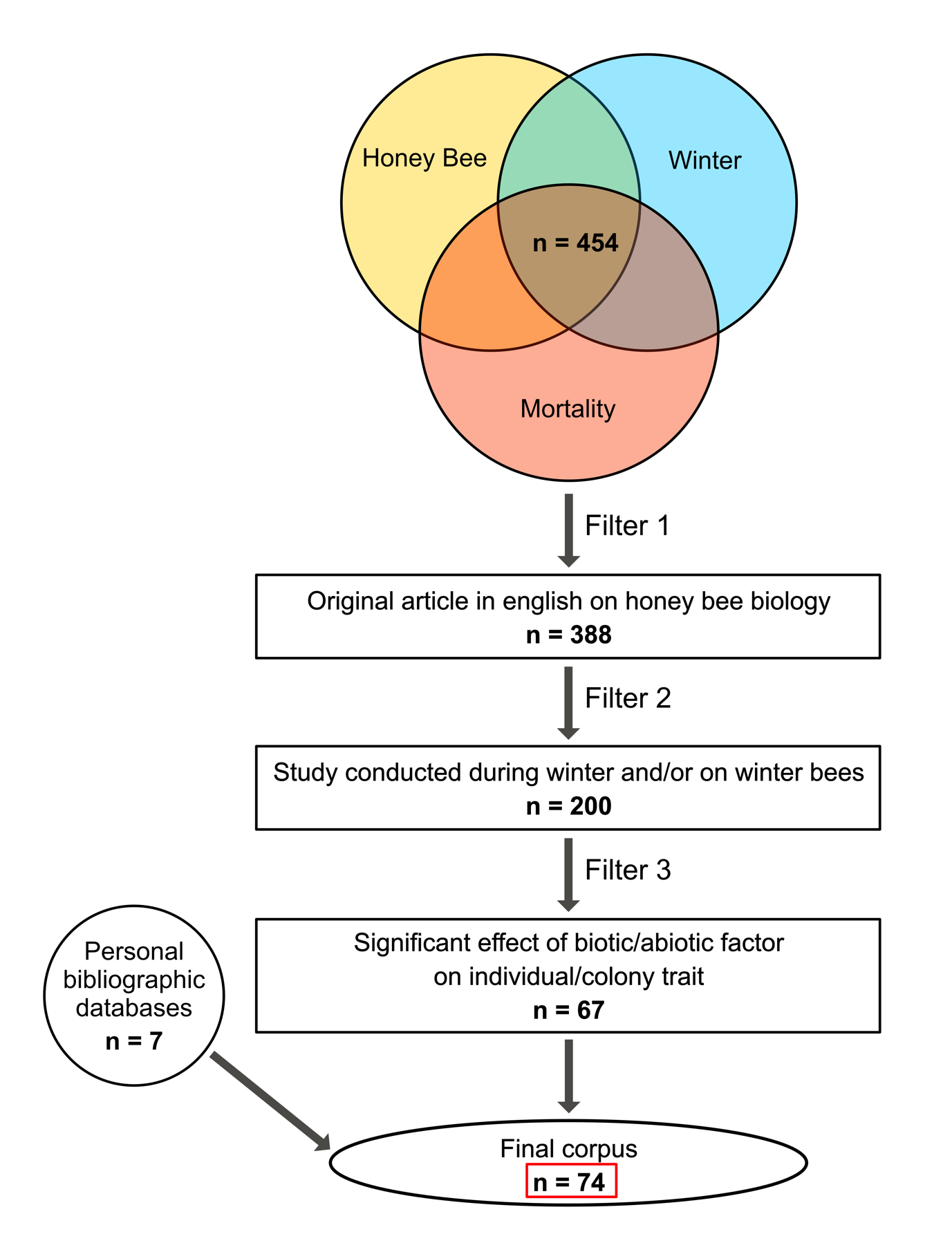


**Figure A.** Diagram of selection criteria applied to Web of Science search article

**Table A.** List of studies found from the systematic synthesis (n=454 studies) and selected for our review (n=74 studies).

| Authors | Article Title | Source Title | Publication Year | Articles origin | Selected | Original article in English on honey bee biology | Study during winter or on winter bees | Significant effect of stressor on a biological trait |
| --- | --- | --- | --- | --- | --- | --- | --- | --- |
| Eischen FA | Overwintering performance of honeybee colonies heavily infest with Acarapis woodi (Rennie) | Apidologie | 1987 | Personal database | Yes | Yes | Yes | Yes |
| Kovac H, Crailsheim K | Lifespan of Apis mellifera carnica(Pollm.) infested by Varroa destructor jacobsoni(Oud.) in relation to season and extent of infestation. | Journal of Apicultural Research | 1988 | Personal database | Yes | Yes | Yes | Yes |
| Skinner AJ | Impacts of tracheal mites (Acarapis woodi (Rennie)) on the respiration and thermoregulation of overwintering honey bees in a temperate climate. | M.Sc. thesis. The University of Guelph, Guelph, Ontario | 2000 | Personal database | Yes | Yes | Yes | Yes |
| Harrison JF, Camazine S, Marden JH et al | Mite not make it home: tracheal mites reduce the safety margin for oxygen delivery of Xying honeybees | Journal of Experimental Biology | 2001 | Personal database | Yes | Yes | Yes | Yes |
| Alburaki, M., Boutin, S., Mercier, P. L., Loublier, Y., Chagnon, M., and Derome, N | Neonicotinoid-coated Zea mays seeds indirectly affect honeybee performance and pathogen susceptibility in field trials | PLoS One | 2015 | Personal database | Yes | Yes | Yes | Yes |
| Baines, D., Wilton, E., Pawluk, A., deGorter, M., and Chomistek, N | Neonicotinoids act like endocrine disrupting chemicals in newly-emerged bees and winter bees | Scientific Reports | 2017 | Personal database | Yes | Yes | Yes | Yes |
| Meikle G. William, Milagra Weiss, Patrick W. Maes, William Fitz, Lucy A.Snyder, Tim Sheehan, Brendon M. Mott, Kirk E. Anderson | Internal hive temperature as a means of monitoring honey bee colony health in a migratory beekeeping management operation before and during winter | Apidologie | 2017 | Personal database | Yes | Yes | Yes | Yes |
| OTIS, GW; SCOTTDUPREE, CD | EFFECTS OF ACARAPIS-WOODI ON OVERWINTERED COLONIES OF HONEY-BEES (HYMENOPTERA, APIDAE) IN NEW-YORK | JOURNAL OF ECONOMIC ENTOMOLOGY | 1992 | WOS research | Yes | Yes | Yes | Yes |
| Martin, SJ | The role of Varroa and viral pathogens in the collapse of honeybee colonies: a modelling approach | JOURNAL OF APPLIED ECOLOGY | 2001 | WOS research | Yes | Yes | Yes | Yes |
| Murilhas, AM | Varroa destructor infestation impact on Apis mellifera carnica capped worker brood production, bee population and honey storage in a Mediterranean climate | APIDOLOGIE | 2002 | WOS research | Yes | Yes | Yes | Yes |
| Decourtye, A; Lacassie, E; Pham-Delègue, MH | Learning performances of honeybees (Apis mellifera L) are differentially affected by imidacloprid according to the season | PEST MANAGEMENT SCIENCE | 2003 | WOS research | Yes | Yes | Yes | Yes |
| Amdam, GV; Hartfelder, K; Norberg, K; Hagen, A; Omholt, SW | Altered physiology in worker honey bees (Hymenoptera: Apidae) infested with the mite Varroa destructor (Acari: Varroidae):: A factor in colony loss during overwintering? | JOURNAL OF ECONOMIC ENTOMOLOGY | 2004 | WOS research | Yes | Yes | Yes | Yes |
| Dodologlu, A; Dülger, C; Genc, F | Colony condition and bee behaviour in honey bees (Apis mellifera) housed in wooden or polystyrene hives and fed 'bee cake' or syrup | JOURNAL OF APICULTURAL RESEARCH | 2004 | WOS research | Yes | Yes | Yes | Yes |
| Currie, RW; Tahmasbi, GH | The ability of high- and low-grooming lines of honey bees to remove the parasitic mite Varroa destructor is affected by environmental conditions | CANADIAN JOURNAL OF ZOOLOGY | 2008 | WOS research | Yes | Yes | Yes | Yes |
| Higes, M; Martín-Hernández, R; Botías, C; Bailón, EG; González-Porto, AV; Barrios, L; del Nozal, MJ; Bernal, JL; Jiménez, JJ; Palencia, PG; Meana, A | How natural infection by Nosema ceranae causes honeybee colony collapse | ENVIRONMENTAL MICROBIOLOGY | 2008 | WOS research | Yes | Yes | Yes | Yes |
| Highfield, AC; El Nagar, A; Mackinder, LCM; Noël, LMLJ; Hall, MJ; Martin, SJ; Schroeder, DC | Deformed Wing Virus Implicated in Overwintering Honeybee Colony Losses | APPLIED AND ENVIRONMENTAL MICROBIOLOGY | 2009 | WOS research | Yes | Yes | Yes | Yes |
| Eberl, HJ; Frederick, MR; Kevan, PG | IMPORTANCE OF BROOD MAINTENANCE TERMS IN SIMPLE MODELS OF THE HONEYBEE - VARROA DESTRUCTOR ACUTE BEE PARALYSIS VIRUS COMPLEX | ELECTRONIC JOURNAL OF DIFFERENTIAL EQUATIONS | 2010 | WOS research | Yes | Yes | Yes | Yes |
| Schäfer, MO; Ritter, W; Pettis, JS; Neumann, P | Concurrent Parasitism Alters Thermoregulation in Honey Bee (Hymenoptera: Apidae) Winter Clusters | ANNALS OF THE ENTOMOLOGICAL SOCIETY OF AMERICA | 2011 | WOS research | Yes | Yes | Yes | Yes |
| Dainat, B; Evans, JD; Chen, YP; Gauthier, L; Neumann, P | Dead or Alive: Deformed Wing Virus and Varroa destructor Reduce the Life Span of Winter Honeybees | APPLIED AND ENVIRONMENTAL MICROBIOLOGY | 2012 | WOS research | Yes | Yes | Yes | Yes |
| van Dooremalen, C; Gerritsen, L; Cornelissen, B; van der Steen, JJM; van Langevelde, F; Blacquière, T | Winter Survival of Individual Honey Bees and Honey Bee Colonies Depends on Level of Varroa destructor Infestation | PLOS ONE | 2012 | WOS research | Yes | Yes | Yes | Yes |
| Toplak, I; Ciglenecki, UJ; Aronstein, K; Gregorc, A | Chronic Bee Paralysis Virus and Nosema ceranae Experimental Co-Infection of Winter Honey Bee Workers (Apis mellifera L.) | VIRUSES-BASEL | 2013 | WOS research | Yes | Yes | Yes | Yes |
| Andrearczyk, S; Kadhim, MJ; Knaga, S | Influence of a probiotic on the mortality, sugar syrup ingestion and infection of honeybees with Nosema spp. under laboratory assessment | MEDYCYNA WETERYNARYJNA-VETERINARY MEDICINE-SCIENCE AND PRACTICE | 2014 | WOS research | Yes | Yes | Yes | Yes |
| Hatjina, F; Costa, C; Büchler, R; Uzunov, A; Drazic, M; Filipi, J; Charistos, L; Ruottinen, L; Andonov, S; Meixner, MD; Bienkowska, M; Gerula, D; Panasiuk, B; Le Conte, Y; Wilde, J; Berg, S; Bouga, M; Dyrba, W; Kiprijanovska, H; Korpela, S; Kryger, P; Lodesani, M; Pechhacker, H; Petrov, P; Kezic, N | Population dynamics of European honey bee genotypes under different environmental conditions | JOURNAL OF APICULTURAL RESEARCH | 2014 | WOS research | Yes | Yes | Yes | Yes |
| Lu, CS; Warchol, KM; Callahan, RA | Sub-lethal exposure to neonicotinoids impaired honey bees winterization before proceeding to colony collapse disorder | BULLETIN OF INSECTOLOGY | 2014 | WOS research | Yes | Yes | Yes | Yes |
| Bahreini, R; Currie, RW | The Potential of Bee-Generated Carbon Dioxide for Control of Varroa Mite (Mesostigmata: Varroidae) in Indoor Overwintering Honey bee (Hymenoptera: Apidae) Colonies | JOURNAL OF ECONOMIC ENTOMOLOGY | 2015 | WOS research | Yes | Yes | Yes | Yes |
| Bahreini, R; Currie, RW | Influence of Honey Bee Genotype and Wintering Method on Wintering Performance of Varroa destructor (Parasitiformes: Varroidae)-Infected Honey Bee (Hymenoptera: Apidae) Colonies in a Northern Climate | JOURNAL OF ECONOMIC ENTOMOLOGY | 2015 | WOS research | Yes | Yes | Yes | Yes |
| Desai, SD; Currie, RW | Genetic diversity within honey bee colonies affects pathogen load and relative virus levels in honey bees, Apis mellifera L | BEHAVIORAL ECOLOGY AND SOCIOBIOLOGY | 2015 | WOS research | Yes | Yes | Yes | Yes |
| Ratti, V; Kevan, PG; Eberl, HJ | A Mathematical Model of the Honeybee-Varroa destructor-Acute Bee Paralysis Virus System with Seasonal Effects | BULLETIN OF MATHEMATICAL BIOLOGY | 2015 | WOS research | Yes | Yes | Yes | Yes |
| Steinmann, N; Corona, M; Neumann, P; Dainat, B | Overwintering Is Associated with Reduced Expression of Immune Genes and Higher Susceptibility to Virus Infection in Honey Bees | PLOS ONE | 2015 | WOS research | Yes | Yes | Yes | Yes |
| DeGrandi-Hoffman, G; Chen, YP; Rivera, R; Carroll, M; Chambers, M; Hidalgo, G; de Jong, EW | Honey bee colonies provided with natural forage have lower pathogen loads and higher overwinter survival than those fed protein supplements | APIDOLOGIE | 2016 | WOS research | Yes | Yes | Yes | Yes |
| Desai, SD; Currie, RW | Effects of Wintering Environment and Parasite-Pathogen Interactions on Honey Bee Colony Loss in North Temperate Regions | PLOS ONE | 2016 | WOS research | Yes | Yes | Yes | Yes |
| Dolezal, AG; Carrillo-Tripp, J; Miller, WA; Bonning, BC; Toth, AL | Intensively Cultivated Landscape and Varroa Mite Infestation Are Associated with Reduced Honey Bee Nutritional State | PLOS ONE | 2016 | WOS research | Yes | Yes | Yes | Yes |
| Hesketh, H; Lahive, E; Horton, AA; Robinson, AG; Svendsen, C; Rortais, A; Dorne, JL; Baas, J; Spurgeon, DJ; Heard, MS | Extending standard testing period in honeybees to predict lifespan impacts of pesticides and heavy metals using dynamic energy budget modelling | SCIENTIFIC REPORTS | 2016 | WOS research | Yes | Yes | Yes | Yes |
| Toomemaa, K; Mänd, M; Williams, IH | Wintering of honey bee colonies in cylindrical nest cavities versus oblong box-hives in a North European climate | JOURNAL OF APICULTURAL RESEARCH | 2016 | WOS research | Yes | Yes | Yes | Yes |
| Wegener, J; Ruhnke, H; Scheller, K; Mispagel, S; Knollmann, U; Kamp, G; Bienefeld, K | Pathogenesis of varroosis at the level of the honey bee (Apis mellifera) colony | JOURNAL OF INSECT PHYSIOLOGY | 2016 | WOS research | Yes | Yes | Yes | Yes |
| Alaux, C; Allier, F; Decourtye, A; Odoux, JF; Tamic, T; Chabirand, M; Delestra, E; Decugis, F; Le Conte, Y; Henry, M | A 'Landscape physiology' approach for assessing bee health highlights the benefits of floral landscape enrichment and semi-natural habitats | SCIENTIFIC REPORTS | 2017 | WOS research | Yes | Yes | Yes | Yes |
| Sánchez-Bayo, F; Belzunces, L; Bonmatin, JM | Lethal and sublethal effects, and incomplete clearance of ingested imidacloprid in honey bees (Apis mellifera) | ECOTOXICOLOGY | 2017 | WOS research | Yes | Yes | Yes | Yes |
| Stalidzans, E; Zacepins, A; Kviesis, A; Brusbardis, V; Meitalovs, J; Paura, L; Bulipopa, N; Liepniece, M | Dynamics of Weight Change and Temperature of Apis mellifera (Hymenoptera: Apidae) Colonies in a Wintering Building With Controlled Temperature | JOURNAL OF ECONOMIC ENTOMOLOGY | 2017 | WOS research | Yes | Yes | Yes | Yes |
| Muñoz-Capponi, EA; Silva-Aguayo, G; Rodríguez-Maciel, JC; Rondanelli-ReYes, MJ | Sublethal exposure to fipronil affects the morphology and development of honey bees, Apis mellifera | BULLETIN OF INSECTOLOGY | 2018 | WOS research | Yes | Yes | Yes | Yes |
| Ptaszynska, AA; Gancarz, M; Hurd, PJ; Borsuk, G; Wiacek, D; Nawrocka, A; Strachecka, A; Zaluski, D; Paleolog, J | Changes in the bioelement content of summer and winter western honeybees (Apis mellifera) induced by Nosema ceranae infection | PLOS ONE | 2018 | WOS research | Yes | Yes | Yes | Yes |
| Dolezal, AG; St Clair, AL; Zhang, G; Toth, AL; O'Neal, ME | Native habitat mitigates feast-famine conditions faced by honey bees in an agricultural landscape | PROCEEDINGS OF THE NATIONAL ACADEMY OF SCIENCES OF THE UNITED STATES OF AMERICA | 2019 | WOS research | Yes | Yes | Yes | Yes |
| Meikle, WG; Corby-Harris, V; Carroll, MJ; Weiss, M; Snyder, LA; Meador, CAD; Beren, E; Brown, N | Exposure to sublethal concentrations of methoxyfenozide disrupts honey bee colony activity and thermoregulation | PLOS ONE | 2019 | WOS research | Yes | Yes | Yes | Yes |
| Quintana, S; Brasesco, C; Negri, P; Marin, M; Pagnuco, I; Szawarski, N; Reynaldi, F; Larsen, A; Eguaras, M; Maggi, M | Up-regulated pathways in response to Deformed Wing Virus infection in Apis mellifera (Hymenoptera: Apidae) | REVISTA DE LA SOCIEDAD ENTOMOLOGICA ARGENTINA | 2019 | WOS research | Yes | Yes | Yes | Yes |
| Requier, F; Rome, Q; Chiron, G; Decante, D; Marion, S; Menard, M; Muller, F; Villemant, C; Henry, M | Predation of the invasive Asian hornet affects foraging activity and survival probability of honey bees in Western Europe | JOURNAL OF PEST SCIENCE | 2019 | WOS research | Yes | Yes | Yes | Yes |
| Shumkova, R; Zhelyazkova, I; Lazarov, S | Application of stimulating products in autumn feeding and wintering of the bee colonies (Apis mellifera L.) | BULGARIAN JOURNAL OF AGRICULTURAL SCIENCE | 2019 | WOS research | Yes | Yes | Yes | Yes |
| Straub, L; Williams, GR; Vidondo, B; Khongphinitbunjong, K; Retschnig, G; Schneeberger, A; Chantawannakul, P; Dietemann, V; Neumann, P | Neonicotinoids and ectoparasitic mites synergistically impact honeybees | SCIENTIFIC REPORTS | 2019 | WOS research | Yes | Yes | Yes | Yes |
| Szawarski, N; Saez, A; Domínguez, E; Dickson, R; De Matteis, A; Eciolaza, C; Justel, M; Aliano, A; Solar, P; Bergara, I; Pons, C; Bolognesi, A; Carna, G; Garcia, W; Garcia, O; Eguaras, M; Lamattina, L; Maggi, M; Negri, P | Effect of Abscisic Acid (ABA) Combined with Two Different Beekeeping Nutritional Strategies to Confront Overwintering: Studies on Honey Bees' Population Dynamics and Nosemosis | INSECTS | 2019 | WOS research | Yes | Yes | Yes | Yes |
| Abi-Akar, F; Schmolke, A; Roy, C; Galic, N; Hinarejos, S | Simulating Honey Bee Large-Scale Colony Feeding Studies Using the BEEHAVE Model-Part II: Analysis of Overwintering Outcomes | ENVIRONMENTAL TOXICOLOGY AND CHEMISTRY | 2020 | WOS research | Yes | Yes | Yes | Yes |
| Almasri, H; Tavares, DA; Pioz, M; Sen, D; Tchamitchian, S; Cousin, M; Brunet, JL; Belzunces, LP | Mixtures of an insecticide, a fungicide and a herbicide induce high toxicities and systemic physiological disturbances in winter Apis mellifera honey bees | ECOTOXICOLOGY AND ENVIRONMENTAL SAFETY | 2020 | WOS research | Yes | Yes | Yes | Yes |
| Bastiaansen, R; Doelman, A; Van Langevelde, F; Rottschäfer, V | MODELING HONEY BEE COLONIES IN WINTER USING A KELLER-SEGEL MODEL WITH A SIGN-CHANGING CHEMOTACTIC COEFFICIENT | SIAM JOURNAL ON APPLIED MATHEMATICS | 2020 | WOS research | Yes | Yes | Yes | Yes |
| Faita, MR; Cardozo, MM; Amandio, DTT; Orth, AI; Nodari, RO | Glyphosate-based herbicides and Nosema sp. microsporidia reduce honey bee (Apis mellifera L.) survivability under laboratory conditions | JOURNAL OF APICULTURAL RESEARCH | 2020 | WOS research | Yes | Yes | Yes | Yes |
| Lu, CS; Chang, CH; Lemos, B; Zhang, Q; MacIntosh, D | Mitochondrial Dysfunction: A Plausible Pathway for Honeybee Colony Collapse Disorder (CCD) | ENVIRONMENTAL SCIENCE & TECHNOLOGY LETTERS | 2020 | WOS research | Yes | Yes | Yes | Yes |
| Lupi, D; Tremolada, P; Colombo, M; Giacchini, R; Benocci, R; Parenti, P; Parolini, M; Zambon, G; Vighi, M | Effects of Pesticides and Electromagnetic Fields on Honeybees: A Field Study Using Biomarkers | INTERNATIONAL JOURNAL OF ENVIRONMENTAL RESEARCH | 2020 | WOS research | Yes | Yes | Yes | Yes |
| Meikle, WG; Weiss, M; Beren, E | Landscape factors influencing honey bee colony behavior in Southern California commercial apiaries | SCIENTIFIC REPORTS | 2020 | WOS research | Yes | Yes | Yes | Yes |
| Sabo, R; Kopcakova, A; Hamarova, L; Maruscakova, IC; Mudronova, D; Sabova, L; Javorsky, P; Legath, J | Sublethal effects of commercial plant protection product containing spores Bacillus amyloliquefaciens QST 713 (formerly subtilis) on winter adult honeybees | APIDOLOGIE | 2020 | WOS research | Yes | Yes | Yes | Yes |
| Saleem, MS; Huang, ZY; Milbrath, MO | Neonicotinoid Pesticides Are More Toxic to Honey Bees at Lower Temperatures: Implications for Overwintering Bees | FRONTIERS IN ECOLOGY AND EVOLUTION | 2020 | WOS research | Yes | Yes | Yes | Yes |
| Tawfik, AI; Ahmed, ZH; Abdel-Rahman, MF; Moustafa, AM | Influence of winter feeding on colony development and the antioxidant system of the honey bee, Apis mellifera | JOURNAL OF APICULTURAL RESEARCH | 2020 | WOS research | Yes | Yes | Yes | Yes |
| Wood, SC; Kozii, IV; de Mattos, IM; Silva, RDM; Klein, CD; Dvylyuk, I; Moshynskyy, I; Epp, T; Simko, E | Chronic High-Dose Neonicotinoid Exposure Decreases Overwinter Survival of Apis mellifera L. | INSECTS | 2020 | WOS research | Yes | Yes | Yes | Yes |
| Hopkins, BK; Chakrabarti, P; Lucas, HM; Sagili, RR; Sheppard, WS | Impacts of Different Winter Storage Conditions on the Physiology of Diutinus Honey Bees (Hymenoptera: Apidae) | JOURNAL OF ECONOMIC ENTOMOLOGY | 2021 | WOS research | Yes | Yes | Yes | Yes |
| Maes, PW; Floyd, AS; Mott, BM; Anderson, KE | Overwintering Honey Bee Colonies: Effect of Worker Age and Climate on the Hindgut Microbiota | INSECTS | 2021 | WOS research | Yes | Yes | Yes | Yes |
| Norrström, N; Niklasson, M; Leidenberger, S | Winter weight loss of different subspecies of honey bee Apis mellifera colonies (Linnaeus, 1758) in southwestern Sweden | PLOS ONE | 2021 | WOS research | Yes | Yes | Yes | Yes |
| Shumkova, R; Balkanska, R; Koynarski, T; Hristov, P | Application of the Natural Products NOZEMAT HERB and NOZEMAT HERB PLUS Can Decrease Honey Bee Colonies Losses during the Winter | DIVERSITY-BASEL | 2021 | WOS research | Yes | Yes | Yes | Yes |
| van Dooremalen, C; van Langevelde, F | Can Colony Size of Honeybees (Apis mellifera) Be Used as Predictor for Colony Losses Due to Varroa destructor during Winter? | AGRICULTURE-BASEL | 2021 | WOS research | Yes | Yes | Yes | Yes |
| Brown, A; Rodriguez, V; Pfister, J; Perreten, V; Neumann, P; Retschnig, G | The dose makes the poison: feeding of antibiotic-treated winter honey bees, Apis mellifera, with probiotics and b-vitamins | APIDOLOGIE | 2022 | WOS research | Yes | Yes | Yes | Yes |
| Noordyke, ER; van Santen, E; Ellis, JD | Evaluating the strength of western honey bee (Apis mellifera L.) colonies fed pollen substitutes over winter | JOURNAL OF APPLIED ENTOMOLOGY | 2022 | WOS research | Yes | Yes | Yes | Yes |
| Pal, E; Almasri, H; Paris, L; Diogon, M; Pioz, M; Cousin, M; Sené, D; Tchamitchian, S; Tavares, DA; Delbac, F; Blot, N; Brunet, JL; Belzunces, LP | Toxicity of the Pesticides Imidacloprid, Difenoconazole and Glyphosate Alone and in Binary and Ternary Mixtures to Winter Honey Bees: Effects on Survival and Antioxidative Defenses | TOXICS | 2022 | WOS research | Yes | Yes | Yes | Yes |
| Sabová, L; Maruscáková, IC; Kolenicová, S; Mudronová, D; Holecková, B; Sabo, R; Sobeková, A; Majchrák, T; Ratvaj, M | The adverse effects of synthetic acaricide tau-fluvalinate (tech.) on winter adult honey bees | ENVIRONMENTAL TOXICOLOGY AND PHARMACOLOGY | 2022 | WOS research | Yes | Yes | Yes | Yes |
| Sarioglu-Bozkurt, A; Topal, E; Günes, N; Üces, E; Cornea-Cipcigan, M; Coskun, I; Cuibus, L; Margaoan, R | Changes in Vitellogenin (Vg) and Stress Protein (HSP 70) in Honey Bee (Apis mellifera anatoliaca) Groups under Different Diets Linked with Physico-Chemical, Antioxidant and Fatty and Amino Acid Profiles | INSECTS | 2022 | WOS research | Yes | Yes | Yes | Yes |
| St Clair, AL; Beach, NJ; Dolezal, AG | Honey bee hive covers reduce food consumption and colony mortality during overwintering | PLOS ONE | 2022 | WOS research | Yes | Yes | Yes | Yes |
| Branchiccela, B; Castelli, L; Díaz-Cetti, S; Invernizzi, C; Mendoza, Y; Santos, E; Silva, C; Zunino, P; Antúnez, K | Can pollen supplementation mitigate the impact of nutritional stress on honey bee colonies? | JOURNAL OF APICULTURAL RESEARCH | 2023 | WOS research | Yes | Yes | Yes | Yes |
| Çakmak, I; Kul, B; Ben Abdelkader, F; Çakmak, SS | Effects of temperature adjustment with a heating device in weak honey bee colonies in cold seasons | INTERNATIONAL JOURNAL OF BIOMETEOROLOGY | 2023 | WOS research | Yes | Yes | Yes | Yes |
| Gerula, D | Health Status of Honeybee Colonies Differing in Genetic Intra-Colonial Diversity | JOURNAL OF APICULTURAL SCIENCE | 2023 | WOS research | Yes | Yes | Yes | Yes |
| Hopkins, BK; Long, J; Naeger, NL; Sheppard, WS | Comparison of indoor (refrigerated) versus outdoor winter storage of commercial honey bee, Apis mellifera (Hymenoptera: Apidae), colonies in the Western United States | JOURNAL OF ECONOMIC ENTOMOLOGY | 2023 | WOS research | Yes | Yes | Yes | Yes |
| Kunc, M; Dobes, P; Ward, R; Lee, S; Cegan, R; Dostalková, S; Holusová, K; Hurychová, J; Eliás, S; Pind'áková, E; Cukanová, E; Prodelalová, J; Petrivalsky, M; Danihlík, J; Havlík, J; Hobza, R; Kavanagh, K; Hyrsl, P | Omics-based analysis of honey bee (Apis mellifera) response to Varroa sp. parasitisation and associated factors reveals changes impairing winter bee generation | INSECT BIOCHEMISTRY AND MOLECULAR BIOLOGY | 2023 | WOS research | Yes | Yes | Yes | Yes |
| JAY, SC; HARRIS, L | LOSS AND DRIFTING OF HONEYBEES FROM HIVES MOVED OUTSIDE AFTER INDOOR WINTERING .1. EFFECT OF VARIOUS ENVIRONMENTAL-FACTORS | JOURNAL OF APICULTURAL RESEARCH | 1979 | WOS research | No | Yes | Yes | No |
| VILLA, JD; KOENIGER, N; RINDERER, TE | OVERWINTERING OF AFRICANIZED, EUROPEAN, AND HYBRID HONEY-BEES IN GERMANY | ENVIRONMENTAL ENTOMOLOGY | 1991 | WOS research | No | Yes | Yes | No |
| VARIS, AL; BALL, BV; ALLEN, M | THE INCIDENCE OF PATHOGENS IN HONEY-BEE (APIS-MELLIFERA L) COLONIES IN FINLAND AND GREAT-BRITAIN | APIDOLOGIE | 1992 | WOS research | No | Yes | Yes | No |
| DUFF, SR; FURGALA, B | EVALUATION OF AMITRAZ AND MENTHOL AS AGENTS TO CONTROL HONEY-BEE TRACHEAL MITE INFESTATIONS IN NONMIGRATORY HONEY-BEE COLONIES IN MINNESOTA | AMERICAN BEE JOURNAL | 1993 | WOS research | No | Yes | Yes | No |
| VILLA, JD; RINDERER, TE | COLD ROOM THERMOREGULATION, STORE CONSUMPTION, AND SURVIVAL OF AFRICANIZED AND EUROPEAN HONEY-BEES (APIS-MELLIFERA L) | APIDOLOGIE | 1993 | WOS research | No | Yes | Yes | No |
| ERICKSON, EH; ERICKSON, BJ; WYMAN, JA | EFFECTS ON HONEY-BEES OF INSECTICIDES APPLIED TO SNAP BEANS IN WISCONSIN - CHEMICAL AND BIOTIC FACTORS | JOURNAL OF ECONOMIC ENTOMOLOGY | 1994 | WOS research | No | Yes | Yes | No |
| KRAUS, B; BERG, S | EFFECT OF A LACTIC-ACID TREATMENT DURING WINTER IN TEMPERATE CLIMATE UPON VARROA-JACOBSONI OUD AND THE BEE (APIS-MELLIFERA L) COLONY | EXPERIMENTAL & APPLIED ACAROLOGY | 1994 | WOS research | No | Yes | Yes | No |
| Finley, J; Camazine, S; Frazier, M | The epidemic of honey bee colony losses during the 1995-1996 season | AMERICAN BEE JOURNAL | 1996 | WOS research | No | Yes | Yes | No |
| Pettis, JS; Wilson, WT | Life history of the honey bee tracheal mite (Acari: Tarsonemidae) | ANNALS OF THE ENTOMOLOGICAL SOCIETY OF AMERICA | 1996 | WOS research | No | Yes | Yes | No |
| Frazier, MT; Finley, J; Harkness, W; Rajotte, EG | A sequential sampling scheme for detecting infestation levels of tracheal mites (Heterostigmata: Tarsonemidae) in honey bee (Hymenoptera: Apidae) colonies | JOURNAL OF ECONOMIC ENTOMOLOGY | 2000 | WOS research | No | Yes | Yes | No |
| Faucon, JP; Mathieu, L; Ribiere, M; Martel, AC; Drajnudel, P; Zeggane, S; Aurieres, C; Aubert, MFA | Honey bee winter mortality in France in 1999 and 2000 | BEE WORLD | 2002 | WOS research | No | Yes | Yes | No |
| Gatien, P; Currie, RW | Timing of acaracide treatments for control of low-level populations of Varroa destructor (Acari: Varroidae) and implications for colony performance of honey bees | CANADIAN ENTOMOLOGIST | 2003 | WOS research | No | Yes | Yes | No |
| Szabo, TI; Szabo, DC | The principles of successfully overwintering honey bees: An examination of colony weight losses during ten winters in southern Ontario | AMERICAN BEE JOURNAL | 2003 | WOS research | No | Yes | Yes | No |
| Kochansky, J; Nasr, M | Laboratory studies on the photostability of fumagillin, the active ingredient of Fumidil B | APIDOLOGIE | 2004 | WOS research | No | Yes | Yes | No |
| Bosch, J; Kemp, WP | Effect of pre-wintering and wintering temperature regimes on weight loss, survival, and emergence time in the mason bee Osmia cornuta (Hymenoptera: Megachilidae) | APIDOLOGIE | 2004 | WOS research | No | Yes | Yes | No |
| Elzen, PJ; Westervelt, D; Lucas, R | Formic acid treatment for control of varroa destructor (Mesostigmata: Varroidae) and safety to Apis mellifera (Hymenoptera: Apidae) under southern United States conditions | JOURNAL OF ECONOMIC ENTOMOLOGY | 2004 | WOS research | No | Yes | Yes | No |
| Sammataro, D; Hoffman, GD; Wardell, G; Finley, J; Ostiguy, N | Testing of a combination of control tactics to manage Varroa destructor (Acari: Varroidae) population levels in honey bee (Hymenoptera: Apidae). | INTERNATIONAL JOURNAL OF ACAROLOGY | 2004 | WOS research | No | Yes | Yes | No |
| Underwood, RM; Currie, RW | Indoor winter fumigation of Apis mellifera (Hymenoptera: Apidae) colonies infested with Varroa destructor (Acari: Varroidae) with formic acid is a potential control alternative in northern climates | JOURNAL OF ECONOMIC ENTOMOLOGY | 2004 | WOS research | No | Yes | Yes | No |
| De Guzman, LI; Rinderer, TE; Bigalk, M; Tubbs, H; Bernard, SJ | Russian honey bee (Hymenoptera: Apidae) colonies:: Acarapis woodi (Acari: Tarsonemidae) infestations and overwintering survival | JOURNAL OF ECONOMIC ENTOMOLOGY | 2005 | WOS research | No | Yes | Yes | No |
| Gregorc, A | Efficacy of oxalic acid and apiguard against varroa mites in honeybee (Apis mellifera) colonies | ACTA VETERINARIA BRNO | 2005 | WOS research | No | Yes | Yes | No |
| Underwood, RM; Currie, RW | Effect of concentration and exposure time on treatment efficacy against varroa mites (Acari: Varroidae) during indoor winter fumigation of honey bees (Hymenoptera: Apidae) with formic acid | JOURNAL OF ECONOMIC ENTOMOLOGY | 2005 | WOS research | No | Yes | Yes | No |
| Fries, I; Imdorf, A; Rosenkranz, P | Survival of mite infested (Varroa destructor) honey bee (Apis mellifera) colonies in a Nordic climate | APIDOLOGIE | 2006 | WOS research | No | Yes | Yes | No |
| Underwood, RM; Currie, RW | Effects of release pattern and room ventilation on survival of varroa mites and queens during indoor winter fumigation of honey bee colonies with formic acid | CANADIAN ENTOMOLOGIST | 2007 | WOS research | No | Yes | Yes | No |
| Lindström, A; Korpela, S; Fries, I | The distribution of Paenibacillus larvae spores in adult bees and honey and larval mortality, following the addition of American foulbrood diseased brood or spore-contaminated honey in honey bee (Apis mellifera) colonies | JOURNAL OF INVERTEBRATE PATHOLOGY | 2008 | WOS research | No | Yes | Yes | No |
| Remolina, SC; Hughes, KA | Evolution and mechanisms of long life and high fertility in queen honey bees | AGE | 2008 | WOS research | No | Yes | Yes | No |
| Topolska, G; Gajda, A; Hartwig, A | POLISH HONEY BEE COLONY-LOSS DURING THE WINTER OF 2007/2008 | JOURNAL OF APICULTURAL SCIENCE | 2008 | WOS research | No | Yes | Yes | No |
| Higes, M; Martín-Hernández, R; Garrido-Bailón, E; González-Porto, AV; García-Palencia, P; Meana, A; del Nozal, MJ; Mayo, R; Bernal, JL | Honeybee colony collapse due to Nosema ceranae in professional apiaries | ENVIRONMENTAL MICROBIOLOGY REPORTS | 2009 | WOS research | No | Yes | Yes | No |
| Berthoud, H; Imdorf, A; Haueter, M; Radloff, S; Neumann, P | Virus infections and winter losses of honey bee colonies (Apis mellifera) | JOURNAL OF APICULTURAL RESEARCH | 2010 | WOS research | No | Yes | Yes | No |
| Genersch, E; von der Ohe, W; Kaatz, H; Schroeder, A; Otten, C; Büchler, R; Berg, S; Ritter, W; Mühlen, W; Gisder, S; Meixner, M; Liebig, G; Rosenkranz, P | The German bee monitoring project: a long term study to understand periodically high winter losses of honey bee colonies | APIDOLOGIE | 2010 | WOS research | No | Yes | Yes | No |
| Schäfer, MO; Ritter, W; Pettis, JS; Neumann, P | Winter Losses of Honeybee Colonies (Hymenoptera: Apidae): The Role of Infestations With Aethina tumida (Coleoptera: Nitidulidae) and Varroa destructor (Parasitiformes: Varroidae) | JOURNAL OF ECONOMIC ENTOMOLOGY | 2010 | WOS research | No | Yes | Yes | No |
| Topolska, G; Gajda, A; Pohorecka, K; Bober, A; Kasprzak, S; Skubida, M; Semkiw, P | Winter colony losses in Poland | JOURNAL OF APICULTURAL RESEARCH | 2010 | WOS research | No | Yes | Yes | No |
| Williams, GR; Shutler, D; Rogers, REL | Effects at Nearctic north-temperate latitudes of indoor versus outdoor overwintering on the microsporidium Nosema ceranae and western honey bees (Apis mellifera) | JOURNAL OF INVERTEBRATE PATHOLOGY | 2010 | WOS research | No | Yes | Yes | No |
| Nguyen, BK; Ribière, M; vanEngelsdorp, D; Snoeck, C; Saegerman, C; Kalkstein, AL; Schurr, F; Brostaux, Y; Faucon, JP; Haubruge, E | Effects of honey bee virus prevalence, Varroa destructor load and queen condition on honey bee colony survival over the winter in Belgium | JOURNAL OF APICULTURAL RESEARCH | 2011 | WOS research | No | Yes | Yes | No |
| Pinto, FD; Puker, A; Message, D; Barreto, LMRC | Varroa destructor in Juquitiba, Vale do Ribeira, Southeastern Brazil: Seasonal Effects on the Infestation Rate of Ectoparasitic Mites on Honeybees | SOCIOBIOLOGY | 2011 | WOS research | No | Yes | Yes | No |
| Pohorecka, K; Bober, A; Skubida, M; Zdanska, D | EPIZOOTIC STATUS OF APIARIES WITH MASSIVE LOSSES OF BEE COLONIES (2008-2009) | JOURNAL OF APICULTURAL SCIENCE | 2011 | WOS research | No | Yes | Yes | No |
| Skerl, MIS; Nakrst, M; Zvokelj, L; Gregorc, A | The acaricidal effect of flumethrin, oxalic acid and amitraz against Varroa destructor in honey bee (Apis mellifera carnica) colonies | ACTA VETERINARIA BRNO | 2011 | WOS research | No | Yes | Yes | No |
| Soroker, V; Hetzroni, A; Yakobson, B; David, D; David, A; Voet, H; Slabezki, Y; Efrat, H; Levski, S; Kamer, Y; Klinberg, E; Zioni, N; Inbar, S; Chejanovsky, N | Evaluation of colony losses in Israel in relation to the incidence of pathogens and pests | APIDOLOGIE | 2011 | WOS research | No | Yes | Yes | No |
| Williams, GR; Shutler, D; Little, CM; Burgher-MacLellan, KL; Rogers, REL | The microsporidian Nosema ceranae, the antibiotic Fumagilin-B®, and western honey bee (Apis mellifera) colony strength | APIDOLOGIE | 2011 | WOS research | No | Yes | Yes | No |
| Botías, C; Martín-Hernández, R; Barrios, L; Garrido-Bailón, E; Nanetti, A; Meana, A; Higes, M | Nosema spp. parasitization decreases the effectiveness of acaricide strips (Apivar®) in treating varroosis of honey bee (Apis mellifera iberiensis) colonies | ENVIRONMENTAL MICROBIOLOGY REPORTS | 2012 | WOS research | No | Yes | Yes | No |
| Dainat, B; Evans, JD; Chen, YP; Gauthier, L; Neumann, P | Predictive Markers of Honey Bee Colony Collapse | PLOS ONE | 2012 | WOS research | No | Yes | Yes | No |
| van der Zee, R; Pisa, L; Andonov, S; Brodschneider, R; Charrière, JD; Chlebo, R; Coffey, MF; Crailsheim, K; Dahle, B; Gajda, A; Gray, A; Drazic, MM; Higes, M; Kauko, L; Kence, A; Kence, M; Kezic, N; Kiprijanovska, H; Kralj, J; Kristiansen, P; Hernandez, RM; Mutinelli, F; Nguyen, BK; Otten, C; Özkirim, A; Pernal, SF; Peterson, M; Ramsay, G; Santrac, V; Soroker, V; Topolska, G; Uzunov, A; Vejsnaes, F; Wei, S; Wilkins, S | Managed honey bee colony losses in Canada, China, Europe, Israel and Turkey, for the winters of 2008-9 and 2009-10 | JOURNAL OF APICULTURAL RESEARCH | 2012 | WOS research | No | Yes | Yes | No |
| Botías, C; Martín-Hernández, R; Barrios, L; Meana, A; Higes, M | Nosema spp. infection and its negative effects on honey bees (Apis mellifera iberiensis) at the colony level | VETERINARY RESEARCH | 2013 | WOS research | No | Yes | Yes | No |
| Dainat, B; Neumann, P | Clinical signs of deformed wing virus infection are predictive markers for honey bee colony losses | JOURNAL OF INVERTEBRATE PATHOLOGY | 2013 | WOS research | No | Yes | Yes | No |
| Erban, T; Jedelsky, PL; Titera, D | Two-dimensional proteomic analysis of honeybee, Apis mellifera, winter worker hemolymph | APIDOLOGIE | 2013 | WOS research | No | Yes | Yes | No |
| Gätschenberger, H; Azzami, K; Tautz, J; Beier, H | Antibacterial Immune Competence of Honey Bees (Apis mellifera) Is Adapted to Different Life Stages and Environmental Risks | PLOS ONE | 2013 | WOS research | No | Yes | Yes | No |
| Ravoet, J; Maharramov, J; Meeus, I; De Smet, L; Wenseleers, T; Smagghe, G; de Graaf, DC | Comprehensive Bee Pathogen Screening in Belgium Reveals Crithidia mellificae as a New Contributory Factor to Winter Mortality | PLOS ONE | 2013 | WOS research | No | Yes | Yes | No |
| Toomemaa, K; Martin, AJ; Mänd, M; Williams, IH | Determining the amount of water condensed above and below the winter cluster of honey bees in a North-European Climate | JOURNAL OF APICULTURAL RESEARCH | 2013 | WOS research | No | Yes | Yes | No |
| Cutler, GC; Scott-Dupree, CD; Sultan, M; McFarlane, AD; Brewer, L | A large-scale field study examining effects of exposure to clothianidin seed-treated canola on honey bee colony health, development, and overwintering success | PEERJ | 2014 | WOS research | No | Yes | Yes | No |
| Ostiguy, N; Eitzer, B | Overwintered brood comb honey: colony exposure to pesticide residues | JOURNAL OF APICULTURAL RESEARCH | 2014 | WOS research | No | Yes | Yes | No |
| Ucak-Koc, A | Effects of altitude and beehive bottom board type on wintering losses of honeybee colonies under subtropical climatic conditions | SPANISH JOURNAL OF AGRICULTURAL RESEARCH | 2014 | WOS research | No | Yes | Yes | No |
| Al Naggar, Y; Tan, Y; Rutherford, C; Connor, W; Griebel, P; Giesy, JP; Robertson, AJ | Effects of treatments with Apivar® and Thymovar® on V-destructor populations, virus infections and indoor winter survival of Canadian honey bee (Apis mellifera L.) colonies | JOURNAL OF APICULTURAL RESEARCH | 2015 | WOS research | No | Yes | Yes | No |
| Clermont, A; Eickermann, M; Kraus, F; Hoffmann, L; Beyer, M | Correlations between land covers and honey bee colony losses in a country with industrialized and rural regions | SCIENCE OF THE TOTAL ENVIRONMENT | 2015 | WOS research | No | Yes | Yes | No |
| Galbraith, DA; Yang, XY; Niño, EL; Yi, S; Grozinger, C | Parallel Epigenomic and Transcriptomic Responses to Viral Infection in Honey Bees (Apis mellifera) | PLOS PATHOGENS | 2015 | WOS research | No | Yes | Yes | No |
| Negri, P; Maggi, MD; Ramirez, L; De Feudis, L; Szwarski, N; Quintana, S; Eguaras, MJ; Lamattina, L | Abscisic acid enhances the immune response in Apis mellifera and contributes to the colony fitness | APIDOLOGIE | 2015 | WOS research | No | Yes | Yes | No |
| Rademacher, E; Harz, M; Schneider, S | The development of HopGuardA® as a winter treatment against Varroa destructor in colonies of Apis mellifera | APIDOLOGIE | 2015 | WOS research | No | Yes | Yes | No |
| Ravoet, J; De Smet, L; Wenseleers, T; de Graaf, DC | Vertical transmission of honey bee viruses in a Belgian queen breeding program | BMC VETERINARY RESEARCH | 2015 | WOS research | No | Yes | Yes | No |
| van der Zee, R; Gray, A; Pisa, L; de Rijk, T | An Observational Study of Honey Bee Colony Winter Losses and Their Association with Varroa destructor, Neonicotinoids and Other Risk Factors | PLOS ONE | 2015 | WOS research | No | Yes | Yes | No |
| Leza, MM; Miranda-Chueca, MA; Purse, BV | Patterns in Varroa destructor depend on bee host abundance, availability of natural resources, and climate in Mediterranean apiaries | ECOLOGICAL ENTOMOLOGY | 2016 | WOS research | No | Yes | Yes | No |
| Betti, MI; Wahl, LM; Zamir, M | Age structure is critical to the population dynamics and survival of honeybee colonies | ROYAL SOCIETY OPEN SCIENCE | 2016 | WOS research | No | Yes | Yes | No |
| Blazyte-Cereskiene, L; Skrodenyte-Arbaciauskiene, V; Radziute, S; Nedveckyte, I; Buda, V | HONEY BEE INFECTION CAUSED BY NOSEMA SPP. IN LITHUANIA | JOURNAL OF APICULTURAL SCIENCE | 2016 | WOS research | No | Yes | Yes | No |
| Burritt, NL; Foss, NJ; Neeno-Eckwall, EC; Church, JO; Hilger, AM; Hildebrand, JA; Warshauer, DM; Perna, NT; Burritt, JB | Sepsis and Hemocyte Loss in Honey Bees (Apis mellifera) Infected with Serratia marcescens Strain Sicaria | PLOS ONE | 2016 | WOS research | No | Yes | Yes | No |
| Corby-Harris, V; Snyder, L; Meador, CAD; Naldo, R; Mott, B; Anderson, KE | Parasaccharibacter apium, gen. nov., sp nov., Improves Honey Bee ( Hymenoptera: Apidae) Resistance to Nosema | JOURNAL OF ECONOMIC ENTOMOLOGY | 2016 | WOS research | No | Yes | Yes | No |
| Giacobino, A; Molineri, A; Cagnolo, NB; Merke, J; Orellano, E; Bertozzi, E; Masciangelo, G; Pietronave, H; Pacini, A; Salto, C; Signorini, M | Queen replacement: the key to prevent winter colony losses in Argentina | JOURNAL OF APICULTURAL RESEARCH | 2016 | WOS research | No | Yes | Yes | No |
| Jack, CJ; Lucas, HM; Webster, TC; Sagili, RR | Colony Level Prevalence and Intensity of Nosema ceranae in Honey Bees (Apis mellifera L.) | PLOS ONE | 2016 | WOS research | No | Yes | Yes | No |
| Maeda, T; Sakamoto, Y | Tracheal mites, Acarapis woodi, greatly increase overwinter mortality in colonies of the Japanese honeybee, Apis cerana japonica | APIDOLOGIE | 2016 | WOS research | No | Yes | Yes | No |
| Roelandt, S; Méroc, E; Riocreux, F; de Graaf, DC; Nguyen, KB; Brunain, M; Verhoeven, B; Dispas, M; Roels, S; Van der Stede, Y | Belgian honey bee winter mortality during 2012-2013: a case-control study and spatial analysis | JOURNAL OF APICULTURAL RESEARCH | 2016 | WOS research | No | Yes | Yes | No |
| Semkiw, P; Skubida, P | SUITABILITY OF STARCH SYRUPS FOR WINTER FEEDING OF HONEYBEE COLONIES | JOURNAL OF APICULTURAL SCIENCE | 2016 | WOS research | No | Yes | Yes | No |
| Smart, MD; Pettis, JS; Euliss, N; Spivak, MS | Land use in the Northern Great Plains region of the US influences the survival and productivity of honey bee colonies | AGRICULTURE ECOSYSTEMS & ENVIRONMENT | 2016 | WOS research | No | Yes | Yes | No |
| Antúnez, K; Invernizzi, C; Mendoza, Y; vanEngelsdorp, D; Zunino, P | Honeybee colony losses in Uruguay during 2013-2014 | APIDOLOGIE | 2017 | WOS research | No | Yes | Yes | No |
| Bixby, M; Baylis, K; Hoover, SE; Currie, RW; Melathopoulos, AP; Pernal, SF; Foster, LJ; Guarna, MM | A Bio-Economic Case Study of Canadian Honey Bee (Hymenoptera: Apidae) Colonies: Marker-Assisted Selection (MAS) in Queen Breeding Affects Beekeeper Profits | JOURNAL OF ECONOMIC ENTOMOLOGY | 2017 | WOS research | No | Yes | Yes | No |
| Chen, C; Liu, ZG; Luo, YX; Xu, Z; Wang, SH; Zhang, XW; Dai, RG; Gao, JL; Chen, X; Guo, HK; Wang, HH; Tang, J; Shi, W | Managed honeybee colony losses of the Eastern honeybee (Apis cerana) in China (2011-2014) | APIDOLOGIE | 2017 | WOS research | No | Yes | Yes | No |
| Colwell, MJ; Williams, GR; Evans, RC; Shutler, D | Honey bee-collected pollen in agro-ecosystems reveals diet diversity, diet quality, and pesticide exposure | ECOLOGY AND EVOLUTION | 2017 | WOS research | No | Yes | Yes | No |
| Jacques, A; Laurent, M; Ribière-Chabert, M; Saussac, M; Bougeard, S; Budge, GE; Hendrikx, P; Chauzat, MP; de Graaf, D; Méroc, E; Nguyen, BK; Roelandt, S; Roels, S; Van der Stede, Y; Tonnersen, T; Kryger, P; Jaarma, K; Kuus, M; Raie, A; Heinikainen, S; Pelkonen, S; Vähänikkilä, N; Andrieux, C; Ballis, A; Barrieu, G; Bendali, F; Brugoux, C; Franco, S; Fuentes, AM; Joel, A; Layec, Y; Lopez, J; Lozach, A; Malherbe, DL; Mariau, V; Meziani, F; Monod, D; Mutel, S; Oesterle, E; Orlowski, M; Petit, M; Pillu, P; Poret, F; Viry, A; Berg, S; Büchler, R; de Craigher, D; Genersch, E; Kaatz, HH; Meixner, MD; von der Ohe, W; Otten, C; Rosenkranz, P; Schäefer, MO; Schroeder, A; Agianiotaki, E; Arfara, S; Boutsini, S; Giannoulopoulou, M; Hondrou, V; Karipidou, S; Katsaros, D; Katzagiannakis, A; Kiriakopoulos, A; Oureilidis, K; Panteli, A; Pantoleon, F; Papagianni, Z; Papalexiou, E; Perdikaris, S; Prapas, A; Siana, P; Skandalakis, I; Stougiou, D; Tomazinakis, I; Tsali, E; Tseliou, E; Tsiplakidis, A; Tsompanellis, E; Vamvakas, G; Varvarouta, V; Vourvidis, D; Dán, A; Daróczi, G; Láng, M; Papp, M; Paulus, PD; Pupp, E; Szalo, M; Tóth, A; Zséli, S; Bressan, G; Cerrone, A; Formato, G; Granato, A; Lavazza, A; Macellari, P; Marcello, P; Ghittino, C; Maroni, PA; Possidente, R; Mutinelli, F; Nassuato, C; Pintore, A; Ricchiuti, L; Ruocco, L; Salvaggio, A; Troiano, P; Voltini, B; Avsejenko, J; Ciekure, E; Deksne, G; Eglite, I; Granta, R; Olsevski, E; Rodze, I; Stinka, M; Sirutkaityte, R; Siriukaitis, S; Bober, A; Jazdzewski, K; Pohorecka, K; Skubida, M; Zdanska, D; Amador, MRR; Freitas, S; Quintans, S; Santos, PT; Brezinova, N; Brtkova, A; Cuvalová, Z; Filipová, M; Jurovciková, J; Kantiková, M; Kubicová, Z; Papierniková, E; Sulejová, L; Toporcák, J; Ares, CCM; Ariza, J; Berná, SN; Cabeza, NA; Casasempere, CJ; Cid, GC; Corzán, RJM; De Abajo, DMA; Diaz, RR; Esteban, RA; Fernández, SP; Garcia, PA; Gonzalez, BC; Minguez, GO; Onate, ML; Oteiza, OP; Pérez, CI; Plaza, PM; Puy, PDJR; Riol, GR; Romero, GLJ; Soldevilla, YJF; Barrasus, MSI; Soriano, GM; Vigo, LV; Villarta, RJL; Fabricius-Kristiansen, L; Forsgren, E; Brown, M; Budge, G; Grant, R; Marris, G; Powell, M; Wattam, A; Whiting, I; Cauquil, L; Riviere, MP; Garin, E | A pan-European epidemiological study reveals honey bee colony survival depends on beekeeper education and disease control | PLOS ONE | 2017 | WOS research | No | Yes | Yes | No |
| Mendoza, Y; Diaz-Cetti, S; Ramallo, G; Santos, E; Porrini, M; Invernizzi, C | Nosema ceranae Winter Control: Study of the Effectiveness of Different Fumagillin Treatments and Consequences on the Strength of Honey Bee (Hymenoptera: Apidae) Colonies | JOURNAL OF ECONOMIC ENTOMOLOGY | 2017 | WOS research | No | Yes | Yes | No |
| Nai, YS; Chen, TY; Chen, YC; Chen, CT; Chen, BY; Chen, YW | Revealing Pesticide Residues Under High Pesticide Stress in Taiwan's Agricultural Environment Probed by Fresh Honey Bee (Hymenoptera: Apidae) Pollen | JOURNAL OF ECONOMIC ENTOMOLOGY | 2017 | WOS research | No | Yes | Yes | No |
| Natsopoulou, ME; McMahon, DP; Doublet, V; Frey, E; Rosenkranz, P; Paxton, RJ | The virulent, emerging genotype B of Deformed wing virus is closely linked to overwinter honeybee worker loss | SCIENTIFIC REPORTS | 2017 | WOS research | No | Yes | Yes | No |
| Pohorecka, K; Szczesna, T; Witek, M; Miszczak, A; Sikorski, P | THE EXPOSURE OF HONEY BEES TO PESTICIDE RESIDUES IN THE HIVE ENVIRONMENT WITH REGARD TO WINTER COLONY LOSSES | JOURNAL OF APICULTURAL SCIENCE | 2017 | WOS research | No | Yes | Yes | No |
| Brown, P; Newstrom-Lloyd, LE; Foster, BJ; Badger, PH; McLean, JA | Winter 2016 honey bee colony losses in New Zealand | JOURNAL OF APICULTURAL RESEARCH | 2018 | WOS research | No | Yes | Yes | No |
| Guler, A; Ekinci, D; Biyik, S; Garipoglu, AV; Onder, H; Kocaokutgen, H | Effects of Feeding Honey Bees (Hymenoptera: Apidae) With Industrial Sugars Produced by Plants Using Different Photosynthetic Cycles (Carbon C3 and C4) on the Colony Wintering Ability, Lifespan, and Forage Behavior | JOURNAL OF ECONOMIC ENTOMOLOGY | 2018 | WOS research | No | Yes | Yes | No |
| Hernando, MD; Gámiz, V; Gil-Lebrero, S; Rodríguez, I; García-Valcárcel, AI; Cutillas, V; Fernández-Alba, AR; Flores, JM | Viability of honeybee colonies exposed to sunflowers grown from seeds treated with the neonicotinoids thiamethoxam and clothianidin | CHEMOSPHERE | 2018 | WOS research | No | Yes | Yes | No |
| Kuchling, S; Kopacka, I; Kalcher-Sommersguter, E; Schwarz, M; Crailsheim, K; Brodschneider, R | Investigating the role of landscape composition on honey bee colony winter mortality: A long-term analysis | SCIENTIFIC REPORTS | 2018 | WOS research | No | Yes | Yes | No |
| Medina-Flores, CA; Esquivel-Marín, NH; López-Carlos, M; Medina-Cuellar, SE; Aguilera-Soto, JI | Estimation of the loss of honey bee colonies in the altiplano and northern regions of Mexico | ECOSISTEMAS Y RECURSOS AGROPECUARIOS | 2018 | WOS research | No | Yes | Yes | No |
| Nouvian, M; Deisig, N; Reinhard, J; Giurfa, M | Seasonality, alarm pheromone and serotonin: insights on the neurobiology of honeybee defence from winter bees | BIOLOGY LETTERS | 2018 | WOS research | No | Yes | Yes | No |
| Brodschneider, R; Brus, J; Danihlík, J | Comparison of apiculture and winter mortality of honey bee colonies (Apis mellifera) in Austria and Czechia | AGRICULTURE ECOSYSTEMS & ENVIRONMENT | 2019 | WOS research | No | Yes | Yes | No |
| Degrandi-Hoffman, G; Graham, H; Ahumada, F; Smart, M; Ziolkowski, N | The Economics of Honey Bee (Hymenoptera: Apidae) Management and Overwintering Strategies for Colonies Used to Pollinate Almonds | JOURNAL OF ECONOMIC ENTOMOLOGY | 2019 | WOS research | No | Yes | Yes | No |
| Haber, AI; Steinhauer, NA; vanEngelsdorp, D | Use of Chemical and Nonchemical Methods for the Control of Varroa destructor (Acari: Varroidae) and Associated Winter Colony Losses in US Beekeeping Operations | JOURNAL OF ECONOMIC ENTOMOLOGY | 2019 | WOS research | No | Yes | Yes | No |
| Kojic, DK; Purac, JS; Nikolic, TV; Orcic, SM; Vujanovic, D; Ilijevic, K; Vukasinovic, EL; Blagojevic, DP | Oxidative stress and the activity of antioxidative defense enzymes in overwintering honey bees | ENTOMOLOGIA GENERALIS | 2019 | WOS research | No | Yes | Yes | No |
| Morawetz, L; Köglberger, H; Griesbacher, A; Derakhshifar, I; Crailsheim, K; Brodschneider, R; Moosbeckhofer, R | Health status of honey bee colonies (Apis mellifera) and disease-related risk factors for colony losses in Austria | PLOS ONE | 2019 | WOS research | No | Yes | Yes | No |
| Nearman, A; vanEngelsdorp, D | What happened to Colony Collapse Disorder? | BERLINER UND MUNCHENER TIERARZTLICHE WOCHENSCHRIFT | 2019 | WOS research | No | Yes | Yes | No |
| Qin, M; Wang, HF; Liu, ZG; Wang, Y; Zhang, WX; Xu, BH | Changes in cold tolerance during the overwintering period in Apis mellifera ligustica | JOURNAL OF APICULTURAL RESEARCH | 2019 | WOS research | No | Yes | Yes | No |
| Toomemaa, K | The synergistic effect of weak oxalic acid and thymol aqueous solutions on Varroa mites and honey bees | JOURNAL OF APICULTURAL RESEARCH | 2019 | WOS research | No | Yes | Yes | No |
| Zana, B; Geiger, L; Kepner, A; Földes, F; Urbán, P; Herczeg, R; Kemenesi, G; Jakab, F | FIRST MOLECULAR DETECTION OF APIS MELLIFERA FILAMENTOUS VIRUS IN HONEY BEES (APIS MELLIFERA) IN HUNGARY | ACTA VETERINARIA HUNGARICA | 2019 | WOS research | No | Yes | Yes | No |
| Bell, HC; Benavides, JE; Montgomery, CN; Navratil, JRE; Nieh, JC | The novel butenolide pesticide flupyradifurone does not alter responsiveness to sucrose at either acute or chronic short-term field-realistic doses in the honey bee, Apis mellifera | PEST MANAGEMENT SCIENCE | 2020 | WOS research | No | Yes | Yes | No |
| Gajger, IT; Svecnjak, L; Bubalo, D; Zorat, T | Control of Varroa destructor Mite Infestations at Experimental Apiaries Situated in Croatia | DIVERSITY-BASEL | 2020 | WOS research | No | Yes | Yes | No |
| Van Esch, L; De Kok, JL; Janssen, L; Buelens, B; De Smet, L; de Graaf, DC; Engelen, G | Multivariate Landscape Analysis of Honey Bee Winter Mortality in Wallonia, Belgium | ENVIRONMENTAL MODELING & ASSESSMENT | 2020 | WOS research | No | Yes | Yes | No |
| Wang, HF; Liu, CL; Liu, ZG; Wang, Y; Ma, LT; Xu, BH | The different dietary sugars modulate the composition of the gut microbiota in honeybee during overwintering | BMC MICROBIOLOGY | 2020 | WOS research | No | Yes | Yes | No |
| Matthijs, S; De Waele, V; Vandenberge, V; Verhoeven, B; Evers, J; Brunain, M; Saegerman, C; De Winter, PJJ; Roels, S; de Graaf, DC; De Regge, N | Nationwide Screening for Bee Viruses and Parasites in Belgian Honey Bees | VIRUSES-BASEL | 2020 | WOS research | No | Yes | Yes | No |
| Abou-Shaara, HF | GIS analysis to locate more suitable wintering areas for honey bee colonies in agricultural and desert lands | AFRICAN ENTOMOLOGY | 2021 | WOS research | No | Yes | Yes | No |
| Becsi, B; Formayer, H; Brodschneider, R | A biophysical approach to assess weather impacts on honey bee colony winter mortality | ROYAL SOCIETY OPEN SCIENCE | 2021 | WOS research | No | Yes | Yes | No |
| Braga, AR; Freitas, BM; Gomes, DG; Bezerra, ADM; Cazier, JA | Forecasting sudden drops of temperature in pre-overwintering honeybee colonies | BIOSYSTEMS ENGINEERING | 2021 | WOS research | No | Yes | Yes | No |
| Calovi, M; Grozinger, CM; Miller, DA; Goslee, SC | Summer weather conditions influence winter survival of honey bees (Apis mellifera) in the northeastern United States | SCIENTIFIC REPORTS | 2021 | WOS research | No | Yes | Yes | No |
| Medina-Flores, CA; Macías-Macías, JO; Cárdenas, AR; Rivera, AS; Vasquez, HIC; Carrillo-Muro, O; López-Carlos, MA | Honey bee colony losses and associated factors in central-western Mexico in the winters 2016 to 2019 | REVISTA BIO CIENCIAS | 2021 | WOS research | No | Yes | Yes | No |
| Punko, RN; Currie, RW; Nasr, ME; Hoover, SE | Epidemiology of Nosema spp. and the effect of indoor and outdoor wintering on honey bee colony population and survival in the Canadian Prairies | PLOS ONE | 2021 | WOS research | No | Yes | Yes | No |
| Steinhauer, N; VanEngelsdorp, D; Saegerman, C | Prioritizing changes in management practices associated with reduced winter honey bee colony losses for US beekeepers | SCIENCE OF THE TOTAL ENVIRONMENT | 2021 | WOS research | No | Yes | Yes | No |
| Stojanov, DP; Dimitrov, L; Danihlík, J; Uzunov, A; Golubovski, M; Andonov, S; Brodschneider, R | Direct Economic Impact Assessment of Winter Honeybee Colony Losses in Three European Countries | AGRICULTURE-BASEL | 2021 | WOS research | No | Yes | Yes | No |
| Anderson, KE; Maes, P | Social microbiota and social gland gene expression of worker honey bees by age and climate | SCIENTIFIC REPORTS | 2022 | WOS research | No | Yes | Yes | No |
| Giacobino, A; Pacini, A; Molineri, A; Bulacio-Cagnolo, N; Merke, J; Orellano, E; Gaggiotti, M; Signorini, M | Impact of nutritional and sanitary management on Apis mellifera colony dynamics and pathogen loads | SPANISH JOURNAL OF AGRICULTURAL RESEARCH | 2022 | WOS research | No | Yes | Yes | No |
| Hernandez, J; Hattendorf, J; Aebi, A; Dietemann, V | Compliance with recommended Varroa destructor treatment regimens improves the survival of honey bee colonies over winter | RESEARCH IN VETERINARY SCIENCE | 2022 | WOS research | No | Yes | Yes | No |
| Johannesen, J; Wöhl, S; Berg, S; Otten, C | Annual Fluctuations in Winter Colony Losses of Apis mellifera L. Are Predicted by Honey Flow Dynamics of the Preceding Year | INSECTS | 2022 | WOS research | No | Yes | Yes | No |
| Li, LH; Lu, CQ; Hong, W; Zhu, YP; Lu, YT; Wang, Y; Xu, BH; Liu, SP | Analysis of temperature characteristics for overwintering bee colonies based on long-term monitoring data | COMPUTERS AND ELECTRONICS IN AGRICULTURE | 2022 | WOS research | No | Yes | Yes | No |
| Mazur, ED; Czopowicz, M; Gajda, AM | Two Faces of the Screened Bottom Boards-An Ambiguous Influence on the Honey Bee Winter Colony Loss Rate | INSECTS | 2022 | WOS research | No | Yes | Yes | No |
| Mendoza, Y; Santos, E; Clavijo-Baquett, S; Invernizzi, C | A Reciprocal Transplant Experiment Confirmed Mite-Resistance in a Honey Bee Population from Uruguay | VETERINARY SCIENCES | 2022 | WOS research | No | Yes | Yes | No |
| Onayemi, SO; Hopkins, BK; Sheppard, WS | Elevated CO2 Increases Overwintering Mortality of Varroa destructor (Mesostigmata: Varroidae) in Honey Bee (Hymenoptera: Apidae) Colonies | JOURNAL OF ECONOMIC ENTOMOLOGY | 2022 | WOS research | No | Yes | Yes | No |
| Retschnig, G; Straub, L; Neumann, P | Conflicting selection on workers underlies global honey bee colony losses | JOURNAL OF APICULTURAL RESEARCH | 2022 | WOS research | No | Yes | Yes | No |
| Sainsbury, J; Nemeth, TE; Baldo, M; Jochym, M; Felman, C; Goodwin, M; Lumsden, M; Pattemore, D; Jeanplong, F | Marker assisted selection for Varroa destructor resistance in New Zealand honey bees | PLOS ONE | 2022 | WOS research | No | Yes | Yes | No |
| Stahlmann-Brown, P; Hall, RJ; Pragert, H; Robertson, T | Varroa Appears to Drive Persistent Increases in New Zealand Colony Losses | INSECTS | 2022 | WOS research | No | Yes | Yes | No |
| Barmak, R; Stefanec, M; Hofstadler, DN; Piotet, L; Schönwetter-Fuchs-Schistek, S; Mondada, F; Schmickl, T; Mills, R | A robotic honeycomb for interaction with a honeybee colony | SCIENCE ROBOTICS | 2023 | WOS research | No | Yes | Yes | No |
| Bixby, M; Scarlett, R; Hoover, SE | Winter mortality, diversification, and self-sufficiency affect honey bee (Hymenoptera: Apidae) colony profit in Canada: a model of commercial Alberta beekeepers | JOURNAL OF ECONOMIC ENTOMOLOGY | 2023 | WOS research | No | Yes | Yes | No |
| DeGrandi-Hoffman, G; Corby-Harris, V; Graham, H; Watkins-deJong, E; Chambers, M; Snyder, L | The survival and growth of honey bee (Hymenoptera: Apidae) colonies overwintered in cold storage: the effects of time and colony location | JOURNAL OF ECONOMIC ENTOMOLOGY | 2023 | WOS research | No | Yes | Yes | No |
| El-Sayed, AS; Al-Rajhi, MAI; Sharobeem, YF | Development of an automatic solar powered honey bee feeding system in a movable multi shelves apiary | JOURNAL OF APICULTURAL RESEARCH | 2023 | WOS research | No | Yes | Yes | No |
| Hernandez, J; Varennes, YD; Aebi, A; Dietemann, V; Kretzschmar, A | Agroecological measures in meadows promote honey bee colony development and winter survival | ECOSPHERE | 2023 | WOS research | No | Yes | Yes | No |
| Kagiali, E; Kokoli, M; Vardakas, P; Goras, G; Hatjina, F; Patalano, S | Four-Year Overview of Winter Colony Losses in Greece: Citizen Science Evidence That Transitioning to Organic Beekeeping Practices Reduces Colony Losses | INSECTS | 2023 | WOS research | No | Yes | Yes | No |
| Kandel, M; Paxton, RJ; Al Naggar, Y | Nationwide Screening for Bee Viruses in Apis mellifera Colonies in Egypt | INSECTS | 2023 | WOS research | No | Yes | Yes | No |
| Kohl, PL; Rutschmann, B; Sikora, LG; Wimmer, N; Zahner, V; D'Alvise, P; Hasselmann, M; Steffan-Dewenter, I | Parasites, depredators, and limited resources as potential drivers of winter mortality of feral honeybee colonies in German forests | OECOLOGIA | 2023 | WOS research | No | Yes | Yes | No |
| Punko, RN; Currie, RW; Nasr, ME; Hoover, SE | Effect of Fumagilin-B treatment timing on nosema (Vairimorpha spp.; Microspora: Nosematidae) abundance and honey bee (Hymenoptera: Apidae) colonies under winter management in the Canadian Prairies | JOURNAL OF ECONOMIC ENTOMOLOGY | 2023 | WOS research | No | Yes | Yes | No |
| Richardson, RT; Conflitti, IM; Labuschagne, RS; Hoover, SE; Currie, RW; Giovenazzo, P; Guarna, MM; Pernal, SF; Foster, LJ; Zayed, A | Land use changes associated with declining honey bee health across temperate North America | ENVIRONMENTAL RESEARCH LETTERS | 2023 | WOS research | No | Yes | Yes | No |
| Schüler, V; Liu, YC; Gisder, S; Horchler, L; Groth, D; Genersch, E | Significant, but not biologically relevant: Nosema ceranae infections and winter losses of honey bee colonies | COMMUNICATIONS BIOLOGY | 2023 | WOS research | No | Yes | Yes | No |
| Scott, A; Hassler, E; Formato, G; Rünzel, MAS; Wilkes, J; Hassan, A; Cazier, J | Data mining hive inspections: more frequently inspected honey bee colonies have higher over-winter survival rates | JOURNAL OF APICULTURAL RESEARCH | 2023 | WOS research | No | Yes | Yes | No |
| Van Espen, M; Williams, JH; Alves, F; Hung, Y; de Graaf, DC; Verbeke, W | Beekeeping in Europe facing climate change: A mixed methods study on perceived impacts and the need to adapt according to stakeholders and beekeepers | SCIENCE OF THE TOTAL ENVIRONMENT | 2023 | WOS research | No | Yes | Yes | No |
| Staveley, JP; Law, SA; Fairbrother, A; Menzie, CA | A Causal Analysis of Observed Declines in Managed Honey Bees (Apis mellifera) | HUMAN AND ECOLOGICAL RISK ASSESSMENT | 2014 | WOS research | No | Yes | Yes | No |
| Clermont, A; Pasquali, M; Eickermann, M; Kraus, F; Hoffmann, L; Beyer, M | VIRUS STATUS, VARROA LEVELS, AND SURVIVAL OF 20 MANAGED HONEY BEE COLONIES MONITORED IN LUXEMBOURG BETWEEN THE SUMMER OF 2011 AND THE SPRING OF 2013 | JOURNAL OF APICULTURAL SCIENCE | 2015 | WOS research | No | Yes | Yes | No |
| Kielmanowicz, MG; Inberg, A; Lerner, IM; Golani, Y; Brown, N; Turner, CL; HaYes, GJR; Ballam, JM | Prospective Large-Scale Field Study Generates Predictive Model Identifying Major Contributors to Colony Losses | PLOS PATHOGENS | 2015 | WOS research | No | Yes | Yes | No |
| Klassen, SS; VanBlyderveen, W; Eccles, L; Kelly, PG; Borges, D; Goodwin, PH; Petukhova, T; Wang, Q; Guzman-Novoa, E | Nosema ceranae Infections in Honey Bees (Apis mellifera) Treated with Pre/Probiotics and Impacts on Colonies in the Field | VETERINARY SCIENCES | 2021 | WOS research | No | Yes | Yes | No |
| CALDERONE, NW; FONDRK, MK | SELECTION FOR HIGH AND LOW, COLONY WEIGHT-GAIN IN THE HONEY-BEE, APIS-MELLIFERA, USING SELECTED QUEENS AND RANDOM MALES | APIDOLOGIE | 1991 | WOS research | No | Yes | No | - |
| DUFF, SR; FURGALA, B | SOME EFFECTS OF MENTHOL ON HONEY-BEE TRACHEAL MITE INFESTATIONS IN NONMIGRATORY HONEY-BEE COLONIES IN MINNESOTA | AMERICAN BEE JOURNAL | 1991 | WOS research | No | Yes | No | - |
| PETTIS, JS; WILSON, WT; SHIMANUKI, H; TEEL, PD | FLUVALINATE TREATMENT OF QUEEN AND WORKER HONEY-BEES (APIS-MELLIFERA L) AND EFFECTS ON SUBSEQUENT MORTALITY, QUEEN ACCEPTANCE AND SUPERSEDURE | APIDOLOGIE | 1991 | WOS research | No | Yes | No | - |
| KORPELA, S; AARHUS, A; FRIES, I; HANSEN, H | VARROA-JACOBSONI OUD IN COLD CLIMATES - POPULATION-GROWTH, WINTER MORTALITY AND INFLUENCE ON THE SURVIVAL OF HONEY-BEE COLONIES | JOURNAL OF APICULTURAL RESEARCH | 1992 | WOS research | No | Yes | No | - |
| RUTTNER, F; HANEL, H | ACTIVE DEFENSE AGAINST VARROA MITES IN A CARNIOLAN STRAIN OF HONEYBEE (APIS-MELLIFERA-CARNICA POLLMANN) | APIDOLOGIE | 1992 | WOS research | No | Yes | No | - |
| Loper, GM | Over-winter losses of feral honey bee colonies in southern Arizona 1992-1997 | AMERICAN BEE JOURNAL | 1997 | WOS research | No | Yes | No | - |
| Korpela, S | Pest status and incidence of the honey bee tracheal mite in Finland | AGRICULTURAL AND FOOD SCIENCE IN FINLAND | 1998 | WOS research | No | Yes | No | - |
| Meled, M; Thrasyvoulou, A; Belzunces, LP | Seasonal variations in susceptibility of Apis mellifera to the synergistic action of prochloraz and deltamethrin | ENVIRONMENTAL TOXICOLOGY AND CHEMISTRY | 1998 | WOS research | No | Yes | No | - |
| Békési, L; Ball, BV; Dobos-Kovács, M; Bakonyi, T; Rusvai, M | Occurrence of acute paralysis virus of the honey bee (Apis mellifera) in a Hungarian apiary infested with the parasitic mite Varroa jacobsoni | ACTA VETERINARIA HUNGARICA | 1999 | WOS research | No | Yes | No | - |
| Pratt, SC | Optimal timing of comb construction by honeybee (Apis mellifera) colonies:: a dynamic programming model and experimental tests | BEHAVIORAL ECOLOGY AND SOCIOBIOLOGY | 1999 | WOS research | No | Yes | No | - |
| Baxter, JR; Ellis, MD; Wilson, WT | Field evaluaton of Apistan® and five candidate compounds for parasitic mite control in honey bees | AMERICAN BEE JOURNAL | 2000 | WOS research | No | Yes | No | - |
| Amir, OG; Peveling, R | Effect of triflumuron on brood development and colony survival of free-flying honeybee, Apis mellifera L. | JOURNAL OF APPLIED ENTOMOLOGY | 2004 | WOS research | No | Yes | No | - |
| Faucon, JP; Aurières, C; Drajnudel, P; Mathieu, L; Ribière, M; Martel, AC; Zeggane, S; Chauzat, MP; Aubert, MFA | Experimental study on the toxicity of imidacloprid given in syrup to honey bee (Apis mellifera) colonies | PEST MANAGEMENT SCIENCE | 2005 | WOS research | No | Yes | No | - |
| Mcmullan, JB; Brown, MJF | Brood pupation temperature affects the susceptibility of honeybees (Apis mellifera) to infestation by tracheal mites (Acarapis woodi) | APIDOLOGIE | 2005 | WOS research | No | Yes | No | - |
| Cutler, GC; Scott-Dupree, CD | Exposure to clothianidin seed-treated canola has no long-term impact on honey bees | JOURNAL OF ECONOMIC ENTOMOLOGY | 2007 | WOS research | No | Yes | No | - |
| Ebert, TA; Kevan, PG; Bishop, BL; Kevan, SD; Downer, RA | Oral toxicity of essential oils and organic acids fed to honey bees (Apis mellifera) | JOURNAL OF APICULTURAL RESEARCH | 2007 | WOS research | No | Yes | No | - |
| Vanengelsdorp, D; Underwood, R; Caron, D; HaYes, J | An estimate of managed colony losses in the winter of 2006-2007: A report commissioned by the apiary inspectors of America | AMERICAN BEE JOURNAL | 2007 | WOS research | No | Yes | No | - |
| Blanchard, P; Schurr, F; Celle, O; Cougoule, N; Drajnudel, P; Thiéry, R; Faucon, JP; Ribière, M | First detection of Israeli acute paralysis virus (IAPV) in France, a dicistrovirus affecting honeybees (Apis mellifera) | JOURNAL OF INVERTEBRATE PATHOLOGY | 2008 | WOS research | No | Yes | No | - |
| Nielsen, SL; Nicolaisen, M; Kryger, P | Incidence of acute bee paralysis virus, black queen cell virus, chronic bee paralysis virus, deformed wing virus, Kashmir bee virus and sacbrood virus in honey bees (Apis mellifera) in Denmark | APIDOLOGIE | 2008 | WOS research | No | Yes | No | - |
| Ward, KN; Coleman, JL; Clinnin, K; Fahrbach, S; Rueppell, O | Age, caste, and behavior determine the replicative activity of intestinal stem cells in honeybees (Apis mellifera L.) | EXPERIMENTAL GERONTOLOGY | 2008 | WOS research | No | Yes | No | - |
| Burgett, M; Rucker, R; Thurman, W | Honey Bee Colony Mortality in the Pacific Northwest (USA) Winter 2007/2008 | AMERICAN BEE JOURNAL | 2009 | WOS research | No | Yes | No | - |
| Nguyen, BK; Saegerman, C; Pirard, C; Mignon, J; Widart, J; Tuirionet, B; Verheggen, FJ; Berkvens, D; De Pauw, E; Haubruge, E | Does Imidacloprid Seed-Treated Maize Have an Impact on Honey Bee Mortality? | JOURNAL OF ECONOMIC ENTOMOLOGY | 2009 | WOS research | No | Yes | No | - |
| Zheng, HQ; Jin, SH; Hu, FL; Pirk, CWW | Sustainable multiple queen colonies of honey bees, Apis mellifera ligustica | JOURNAL OF APICULTURAL RESEARCH | 2009 | WOS research | No | Yes | No | - |
| Bienefeld, K | Does Breeding in the Honey Bee Result in Higher Winter Losses? | AMERICAN BEE JOURNAL | 2010 | WOS research | No | Yes | No | - |
| Caron, D; Burgett, M; Rucker, R; Thurman, W | Honey Bee Colony Mortality in the Pacific Northwest: Winter 2008/2009 | AMERICAN BEE JOURNAL | 2010 | WOS research | No | Yes | No | - |
| Charrière, JD; Imdorf, A; Koenig, C; Gallmann, S; Kuhn, R | Do sunflowers influence the development of honey bee, Apis mellifera, colonies in areas with diversified crop farming? | JOURNAL OF APICULTURAL RESEARCH | 2010 | WOS research | No | Yes | No | - |
| Chauzat, MP; Carpentier, P; Madec, F; Bougeard, S; Cougoule, N; Drajnudel, P; Clément, MC; Aubert, M; Faucon, JP | The role of infectious agents and parasites in the health of honey bee colonies in France | JOURNAL OF APICULTURAL RESEARCH | 2010 | WOS research | No | Yes | No | - |
| Gajger, IT; Tomljanovic, Z; Petrinec, Z | Monitoring health status of Croatian honey bee colonies and possible reasons for winter losses | JOURNAL OF APICULTURAL RESEARCH | 2010 | WOS research | No | Yes | No | - |
| Gisder, S; Hedtke, K; Möckel, N; Frielitz, MC; Linde, A; Genersch, E | Five-Year Cohort Study of Nosema spp. in Germany: Does Climate Shape Virulence and Assertiveness of Nosema ceranae? | APPLIED AND ENVIRONMENTAL MICROBIOLOGY | 2010 | WOS research | No | Yes | No | - |
| Guzmán-Novoa, E; Eccles, L; Calvete, Y; Mcgowan, J; Kelly, PG; Correa-Benítez, A | Varroa destructor is the main culprit for the death and reduced populations of overwintered honey bee (Apis mellifera) colonies in Ontario, Canada | APIDOLOGIE | 2010 | WOS research | No | Yes | No | - |
| Harris, JL | The effect of requeening in late July on honey bee colony development on the Northern Great Plains of North America after removal from an indoor winter storage facility | JOURNAL OF APICULTURAL RESEARCH | 2010 | WOS research | No | Yes | No | - |
| Ivanova, EN; Petrov, PP | Regional differences in honey bee winter losses in Bulgaria during the period 2006-9 | JOURNAL OF APICULTURAL RESEARCH | 2010 | WOS research | No | Yes | No | - |
| Martin, SJ; Ball, BV; Carreck, NL | Prevalence and persistence of deformed wing virus (DWV) in untreated or acaricide-treated Varroa destructor infested honey bee (Apis mellifera) colonies | JOURNAL OF APICULTURAL RESEARCH | 2010 | WOS research | No | Yes | No | - |
| Mutinelli, F; Sgolastra, F; Gallina, A; Medrzycki, P; Bortolotti, L; Lodesani, M; Porrini, C | A Network for Monitoring Honey Bee Mortality and Colony Losses in Italy as a Part of the APENET Research Project | AMERICAN BEE JOURNAL | 2010 | WOS research | No | Yes | No | - |
| Nguyen, BK; Mignon, J; Laget, D; de Graaf, DC; Jacobs, FJ; vanEngelsdorp, D; Brostaux, Y; Saegerman, C; Haubruge, E | Honey bee colony losses in Belgium during the 2008-9 winter | JOURNAL OF APICULTURAL RESEARCH | 2010 | WOS research | No | Yes | No | - |
| vanEngelsdorp, D; Speybroeck, N; Evans, JD; Nguyen, BK; Mullin, C; Frazier, M; Frazier, J; Cox-Foster, D; Chen, YP; Tarpy, DR; Haubruge, E; Pettis, JS; Saegerman, C | Weighing Risk Factors Associated With Bee Colony Collapse Disorder by Classification and Regression Tree Analysis | JOURNAL OF ECONOMIC ENTOMOLOGY | 2010 | WOS research | No | Yes | No | - |
| Caron, D; Sagili, R | Honey Bee Colony Mortality in the Pacific Northwest: Winter 2009/2010 | AMERICAN BEE JOURNAL | 2011 | WOS research | No | Yes | No | - |
| Di Prisco, G; Zhang, X; Pennacchio, F; Caprio, E; Li, JL; Evans, JD; DeGrandi-Hoffman, G; Hamilton, M; Chen, YP | Dynamics of Persistent and Acute Deformed Wing Virus Infections in Honey Bees, Apis mellifera | VIRUSES-BASEL | 2011 | WOS research | No | Yes | No | - |
| Hawthorne, DJ; Dively, GP | Killing Them with Kindness? In-Hive Medications May Inhibit Xenobiotic Efflux Transporters and Endanger Honey Bees | PLOS ONE | 2011 | WOS research | No | Yes | No | - |
| Hedtke, K; Jensen, PM; Jensen, AB; Genersch, E | Evidence for emerging parasites and pathogens influencing outbreaks of stress-related diseases like chalkbrood | JOURNAL OF INVERTEBRATE PATHOLOGY | 2011 | WOS research | No | Yes | No | - |
| Kojima, Y; Toki, T; Morimoto, T; Yoshiyama, M; Kimura, K; Kadowaki, T | Infestation of Japanese Native Honey Bees by Tracheal Mite and Virus from Non-native European Honey Bees in Japan | MICROBIAL ECOLOGY | 2011 | WOS research | No | Yes | No | - |
| Runckel, C; Flenniken, ML; Engel, JC; Ruby, JG; Ganem, D; Andino, R; DeRisi, JL | Temporal Analysis of the Honey Bee Microbiome Reveals Four Novel Viruses and Seasonal Prevalence of Known Viruses, Nosema, and Crithidia | PLOS ONE | 2011 | WOS research | No | Yes | No | - |
| Farjan, M; Dmitryjuk, M; Lipinski, Z; Biernat-Lopienska, E; Zóltowska, K | Supplementation of the honey bee diet with vitamin C: The effect on the antioxidative system of Apis mellifera carnica brood at different stages | JOURNAL OF APICULTURAL RESEARCH | 2012 | WOS research | No | Yes | No | - |
| Freiberg, M; De Jong, D; Message, D; Cox-Foster, D | First report of sacbrood virus in honey bee (Apis mellifera) colonies in Brazil | GENETICS AND MOLECULAR RESEARCH | 2012 | WOS research | No | Yes | No | - |
| Sugahara, M; Nishimura, Y; Sakamoto, F | Differences in Heat Sensitivity between Japanese Honeybees and Hornets under High Carbon Dioxide and Humidity Conditions inside Bee Balls | ZOOLOGICAL SCIENCE | 2012 | WOS research | No | Yes | No | - |
| Coffey, MF; Breen, J | Efficacy of Apilife Var® and Thymovar® against Varroa destructor as an autumn treatment in a cool climate | JOURNAL OF APICULTURAL RESEARCH | 2013 | WOS research | No | Yes | No | - |
| Francis, RM; Nielsen, SL; Kryger, P | Varroa-Virus Interaction in Collapsing Honey Bee Colonies | PLOS ONE | 2013 | WOS research | No | Yes | No | - |
| Mao, W; Schuler, MA; Berenbaum, MR | Honey constituents up-regulate detoxification and immunity genes in the western honey bee Apis mellifera | PROCEEDINGS OF THE NATIONAL ACADEMY OF SCIENCES OF THE UNITED STATES OF AMERICA | 2013 | WOS research | No | Yes | No | - |
| Münch, D; Kreibich, CD; Amdam, GV | Aging and its modulation in a long-lived worker caste of the honey bee | JOURNAL OF EXPERIMENTAL BIOLOGY | 2013 | WOS research | No | Yes | No | - |
| Pilling, E; Campbell, P; Coulson, M; Ruddle, N; Tornier, I | A Four-Year Field Program Investigating Long-Term Effects of Repeated Exposure of Honey Bee Colonies to Flowering Crops Treated with Thiamethoxam | PLOS ONE | 2013 | WOS research | No | Yes | No | - |
| Ptaszynska, AA; Borsuk, G; Mulenko, W; Olszewski, K | Impact of ethanol on Nosema spp. infected bees | MEDYCYNA WETERYNARYJNA-VETERINARY MEDICINE-SCIENCE AND PRACTICE | 2013 | WOS research | No | Yes | No | - |
| Sammataro, D; Weiss, M | Comparison of productivity of colonies of honey bees, Apis mellifera, supplemented with sucrose or high fructose corn syrup | JOURNAL OF INSECT SCIENCE | 2013 | WOS research | No | Yes | No | - |
| Semkiw, P; Skubida, P; Pohorecka, K | THE AMITRAZ STRIPS EFFICACY IN CONTROL OF VARROA DESTRUCTOR AFTER MANY YEARS APPLICATION OF AMITRAZ IN APIARIES | JOURNAL OF APICULTURAL SCIENCE | 2013 | WOS research | No | Yes | No | - |
| Stevanovic, J; Simeunovic, P; Gajic, B; Lakic, N; Radovic, D; Fries, I; Stanimirovic, Z | Characteristics of Nosema ceranae infection in Serbian honey bee colonies | APIDOLOGIE | 2013 | WOS research | No | Yes | No | - |
| Strauss, U; Human, H; Gauthier, L; Crewe, RM; Dietemann, V; Pirk, CWW | Seasonal prevalence of pathogens and parasites in the savannah honeybee (Apis mellifera scutellata) | JOURNAL OF INVERTEBRATE PATHOLOGY | 2013 | WOS research | No | Yes | No | - |
| vanEngelsdorp, D; Tarpy, DR; Lengerich, EJ; Pettis, JS | Idiopathic brood disease syndrome and queen events as precursors of colony mortality in migratory beekeeping operations in the eastern United States | PREVENTIVE VETERINARY MEDICINE | 2013 | WOS research | No | Yes | No | - |
| Betti, MI; Wahl, LM; Zamir, M | Effects of Infection on Honey Bee Population Dynamics: A Model | PLOS ONE | 2014 | WOS research | No | Yes | No | - |
| Bonoan, RE; Goldman, RR; Wong, PY; Starks, PT | Vasculature of the hive: heat dissipation in the honey bee (Apis mellifera) hive | NATURWISSENSCHAFTEN | 2014 | WOS research | No | Yes | No | - |
| Ferrari, TE | Magnets, magnetic field fluctuations and geomagnetic disturbances impair the homing ability of honey bees (Apis mellifera) | JOURNAL OF APICULTURAL RESEARCH | 2014 | WOS research | No | Yes | No | - |
| Gajger, IT; Kolodziejek, J; Bakonyi, T; Nowotny, N | Prevalence and distribution patterns of seven different honeybee viruses in diseased colonies: a case study from Croatia | APIDOLOGIE | 2014 | WOS research | No | Yes | No | - |
| Giacobino, A; Cagnolo, NB; Merke, J; Orellano, E; Bertozzi, E; Masciangelo, G; Pietronave, H; Salto, C; Signorini, M | Risk factors associated with the presence of Varroa destructor in honey bee colonies from east-central Argentina | PREVENTIVE VETERINARY MEDICINE | 2014 | WOS research | No | Yes | No | - |
| Hristov, Y | REASONS FOR BEE COLONY LOSSES IN BULGARIA AND THEIR RELATION WITH CCD | COMPTES RENDUS DE L ACADEMIE BULGARE DES SCIENCES | 2014 | WOS research | No | Yes | No | - |
| Li, JL; Cornman, RS; Evans, JD; Pettis, JS; Zhao, Y; Murphy, C; Peng, WJ; Wu, J; Hamilton, M; Boncristiani, HF; Zhou, L; Hammond, J; Chen, YP | Systemic Spread and Propagation of a Plant-Pathogenic Virus in European Honeybees, Apis mellifera | MBIO | 2014 | WOS research | No | Yes | No | - |
| Locke, B; Forsgren, E; de Miranda, JR | Increased Tolerance and Resistance to Virus Infections: A Possible Factor in the Survival of Varroa destructor-Resistant Honey Bees (Apis mellifera) | PLOS ONE | 2014 | WOS research | No | Yes | No | - |
| Muli, E; Patch, H; Frazier, M; Frazier, J; Torto, B; Baumgarten, T; Kilonzo, J; Kimani, JN; Mumoki, F; Masiga, D; Tumlinson, J; Grozinger, C | Evaluation of the Distribution and Impacts of Parasites, Pathogens, and Pesticides on Honey Bee (Apis mellifera) Populations in East Africa | PLOS ONE | 2014 | WOS research | No | Yes | No | - |
| Rinderer, TE; Danka, RG; Johnson, S; Bourgeois, AL; Frake, AM; Villa, JD; De Guzman, LI; Harris, JW | Functionality of Varroa-Resistant Honey Bees (Hymenoptera: Apidae) When Used for Western US Honey Production and Almond Pollination | JOURNAL OF ECONOMIC ENTOMOLOGY | 2014 | WOS research | No | Yes | No | - |
| Ryabov, EV; Wood, GR; Fannon, JM; Moore, JD; Bull, JC; Chandler, D; Mead, A; Burroughs, N; Evans, DJ | A Virulent Strain of Deformed Wing Virus (DWV) of Honeybees (Apis mellifera) Prevails after Varroa destructor-Mediated, or In Vitro, Transmission | PLOS PATHOGENS | 2014 | WOS research | No | Yes | No | - |
| Sandrock, C; Tanadini, M; Tanadini, LG; Fauser-Misslin, A; Potts, SG; Neumann, P | Impact of Chronic Neonicotinoid Exposure on Honeybee Colony Performance and Queen Supersedure | PLOS ONE | 2014 | WOS research | No | Yes | No | - |
| Simeunovic, P; Stevanovic, E; Cirkovic, D; Radojicic, S; Lakic, N; Stanisic, L; Stanimirovic, Z | Nosema ceranae and queen age influence the reproduction and productivity of the honey bee colony | JOURNAL OF APICULTURAL RESEARCH | 2014 | WOS research | No | Yes | No | - |
| Wegrzynowicz, P; Gerula, D; Bienkowska, M; Panasiuk, B | CAUSES AND SCALE OF WINTER FLIGHTS IN HONEY BEE (APIS MELLIFERA CARNICA) COLONIES | JOURNAL OF APICULTURAL SCIENCE | 2014 | WOS research | No | Yes | No | - |
| Al Toufailia, H; Scandian, L; Ratnieks, FLW | Towards integrated control of varroa: 2)comparing application methods and doses of oxalic acid on the mortality of phoretic Varroa destructor mites and their honey bee hosts | JOURNAL OF APICULTURAL RESEARCH | 2015 | WOS research | No | Yes | No | - |
| Amiri, E; Meixner, M; Nielsen, SL; Kryger, P | Four Categories of Viral Infection Describe the Health Status of Honey Bee Colonies | PLOS ONE | 2015 | WOS research | No | Yes | No | - |
| Champetier, A; Sumner, DA; Wilen, JE | The Bioeconomics of Honey Bees and Pollination | ENVIRONMENTAL & RESOURCE ECONOMICS | 2015 | WOS research | No | Yes | No | - |
| Dively, GP; Embrey, MS; Kamel, A; Hawthorne, DJ; Pettis, JS | Assessment of Chronic Sublethal Effects of Imidacloprid on Honey Bee Colony Health | PLOS ONE | 2015 | WOS research | No | Yes | No | - |
| Farjan, M; Zóltowska, K; Lipinski, Z; Lopienska-Biernat, E; Dmitryjuk, M | THE EFFECT OF DIETARY VITAMIN C ON CARBOHYDRATE CONCENTRATIONS AND HYDROLASE ACTIVITY, DURING THE DEVELOPMENT OF HONEY BEE WORKER BROOD | JOURNAL OF APICULTURAL SCIENCE | 2015 | WOS research | No | Yes | No | - |
| Giacobino, A; Molineri, A; Cagnolo, NB; Merke, J; Orellano, E; Bertozzi, E; Masciángelo, G; Pietronave, H; Pacini, A; Salto, C; Signorini, M | Risk factors associated with failures of Varroa treatments in honey bee colonies without broodless period | APIDOLOGIE | 2015 | WOS research | No | Yes | No | - |
| Peng, WJ; Li, JL; Zhao, YZ; Chen, YP; Zeng, ZJ | A descriptive study of the prevalence of parasites and pathogens in Chinese black honeybees | PARASITOLOGY | 2015 | WOS research | No | Yes | No | - |
| Rinkevich, FD; Margotta, JW; Pittman, JM; Danka, RG; Tarver, MR; Ottea, JA; Healy, KB | Genetics, Synergists, and Age Affect Insecticide Sensitivity of the Honey Bee, Apis mellifera | PLOS ONE | 2015 | WOS research | No | Yes | No | - |
| Roy, C; Vidal-Naquet, N; Provost, B | A severe sacbrood virus outbreak in a honeybee (Apis mellifera L.) colony: a case report | VETERINARNI MEDICINA | 2015 | WOS research | No | Yes | No | - |
| Scofield, HN; Mattila, HR | Honey Bee Workers That Are Pollen Stressed as Larvae Become Poor Foragers and Waggle Dancers as Adults | PLOS ONE | 2015 | WOS research | No | Yes | No | - |
| Adjlane, N; Dainat, B; Gauthier, L; Dietemann, V | Atypical viral and parasitic pattern in Algerian honey bee subspecies Apis mellifera intermissa and A-m. sahariensis | APIDOLOGIE | 2016 | WOS research | No | Yes | No | - |
| Alayrangues, J; Hotier, L; Massou, I; Bertrand, Y; Armengaud, C | Prolonged effects of in-hive monoterpenoids on the honey bee Apis mellifera | ECOTOXICOLOGY | 2016 | WOS research | No | Yes | No | - |
| Ayton, S; Tomlinson, S; Phillips, RD; Dixon, KW; Withers, PC | Phenophysiological variation of a bee that regulates hive humidity, but not hive temperature | JOURNAL OF EXPERIMENTAL BIOLOGY | 2016 | WOS research | No | Yes | No | - |
| Cavigli, I; Daughenbaugh, KF; Martin, M; Lerch, M; Banner, K; Garcia, E; Brutscher, LM; Flenniken, ML | Pathogen prevalence and abundance in honey bee colonies involved in almond pollination | APIDOLOGIE | 2016 | WOS research | No | Yes | No | - |
| Chaimanee, V; Evans, JD; Chen, YP; Jackson, C; Pettis, JS | Sperm viability and gene expression in honey bee queens (Apis mellifera) following exposure to the neonicotinoid insecticide imidacloprid and the organophosphate acaricide coumaphos | JOURNAL OF INSECT PHYSIOLOGY | 2016 | WOS research | No | Yes | No | - |
| Chauzat, MP; Jacques, A; Laurent, M; Bougeard, S; Hendrikx, P; Ribière-Chabert, M; de Graaf, D; Méroc, E; Nguyen, BK; Roelandt, S; Roels, S; Van der Stede, Y; Tonnersen, T; Kryger, P; Jaarma, K; Kuus, M; Raie, A; Heinikainen, S; Pelkonen, S; Vähänikkilä, N; Andrieux, C; Ballis, A; Barrieu, G; Bendali, F; Brugoux, C; Franco, S; Fuentes, AM; Joel, A; Layec, Y; Lopez, J; Lozach, A; Malherbe-Duluc, L; Mariau, V; Meziani, F; Monod, D; Mutel, S; Oesterle, E; Orlowski, M; Petit, M; Pillu, P; Poret, F; Viry, A; Berg, S; Büchler, R; de Craigher, D; Genersch, E; Kaatz, HH; Meixner, MD; von der Ohe, W; Otten, C; Rosenkranz, P; Schäfer, MO; Schroeder, A; Agianiotaki, E; Arfara, S; Boutsini, S; Giannoulopoulou, M; Hondrou, V; Karipidou, S; Katsaros, D; Katzagiannakis, A; Kiriakopoulos, A; Oureilidis, K; Panteli, A; Pantoleon, F; Papagianni, Z; Papalexiou, E; Perdikaris, S; Prapas, A; Siana, P; Skandalakis, I; Stougiou, D; Tomazinakis, I; Tsali, E; Tseliou, E; Tsiplakidis, A; Tsompanellis, E; Vamvakas, G; Varvarouta, V; Vourvidis, D; Dán, A; Daróczi, G; Láng, M; Papp, M; Paulus, PD; Pupp, E; Szaló, M; Tóth, A; Zséli, S; Bressan, G; Cerrone, A; Formato, G; Granato, A; Lavazza, A; Macellari, P; Marcello, P; Ghittino, C; Maroni, PA; Possidente, R; Mutinelli, F; Nassuato, C; Pintore, A; Ricchiuti, L; Ruocco, L; Salvaggio, A; Troiano, P; Voltini, B; Avsejenko, J; Ciekure, E; Deksne, G; Eglïte, I; Granta, R; OlsEvski, E; Rodze, I; Stinka, M; Sirutkaityte, R; Siriukaitis, S; Bober, A; Jazdzewski, K; Pohorecka, K; Skubida, M; Zdanska, D; Amador, MRR; Freitas, S; Quintans, S; Santos, PT; Brezinová, N; Brtková, A; Cuvalová, Z; Filipová, M; Jurovciková, J; Kantíková, M; Kubicová, Z; Papierniková, E; Sulejová, L; Toporcák, J; Cenador, CMA; Ariza, J; Serna, NB; Núñez, AC; Cascales, JC; González, CC; Ripoll, JMC; Domingo, MAD; Rey, RD; Royo, AE; Somalo, PF; Pascualvaca, PG; Breña, CG; Gonzalez, OM; Oñate, ML; Orradre, PO; Cobo, IP; Pérez, MP; Pitarque, DJRP; Guinea, RR; Gonzalez, LJR; Yanguas, JFS; Barrasús, MSI; López, VL; Rivas, JLV; Fabricius-Kristiansen, L; Forsgren, E; Brown, M; Budge, G; Grant, R; Marris, G; Powell, M; Wattam, A; Whiting, I; Cauquil, L; Garin, E; Rivière, MP | Risk indicators affecting honeybee colony survival in Europe: one year of surveillance | APIDOLOGIE | 2016 | WOS research | No | Yes | No | - |
| Chen, C; Liu, ZG; Pan, Q; Chen, X; Wang, HH; Guo, HK; Liu, SD; Lu, HF; Tian, SL; Li, RQ; Shi, W | Genomic Analyses Reveal Demographic History and Temperate Adaptation of the Newly Discovered Honey Bee Subspecies Apis mellifera sinisxinyuan n. ssp | MOLECULAR BIOLOGY AND EVOLUTION | 2016 | WOS research | No | Yes | No | - |
| Coffey, MF; Breen, J | The efficacy and tolerability of Api-Bioxal® as a winter varroacide in a cool temperate climate | JOURNAL OF APICULTURAL RESEARCH | 2016 | WOS research | No | Yes | No | - |
| Li, W; Evans, JD; Huang, Q; Rodríguez-García, C; Liu, J; Hamilton, M; Grozinger, CM; Webster, TC; Su, S; Chen, YP | Silencing the Honey Bee (Apis mellifera) Naked Cuticle Gene (nkd) Improves Host Immune Function and Reduces Nosema ceranae Infections | APPLIED AND ENVIRONMENTAL MICROBIOLOGY | 2016 | WOS research | No | Yes | No | - |
| Mordecai, GJ; Wilfert, L; Martin, SJ; Jones, IM; Schroeder, DC | Diversity in a honey bee pathogen: first report of a third master variant of the Deformed Wing Virus quasispecies | ISME JOURNAL | 2016 | WOS research | No | Yes | No | - |
| Porrini, C; Mutinelli, F; Bortolotti, L; Granato, A; Laurenson, L; Roberts, K; Gallina, A; Silvester, N; Medrzycki, P; Renzi, T; Sgolastra, F; Lodesani, M | The Status of Honey Bee Health in Italy: Results from the Nationwide Bee Monitoring Network | PLOS ONE | 2016 | WOS research | No | Yes | No | - |
| Reetz, JE; Schulz, W; Seitz, W; Spiteller, M; Zuehlke, S; Armbruster, W; Wallner, K | Uptake of Neonicotinoid Insecticides by Water-Foraging Honey Bees (Hymenoptera: Apidae) Through Guttation Fluid of Winter Oilseed Rape | JOURNAL OF ECONOMIC ENTOMOLOGY | 2016 | WOS research | No | Yes | No | - |
| Schmickl, T; Karsai, I | How regulation based on a common stomach leads to economic optimization of honeybee foraging | JOURNAL OF THEORETICAL BIOLOGY | 2016 | WOS research | No | Yes | No | - |
| Boyle, NK; Sheppard, WS | A scientific note on seasonal levels of pesticide residues in honey bee worker tissues | APIDOLOGIE | 2017 | WOS research | No | Yes | No | - |
| Gisder, S; Schüler, V; Horchler, LL; Groth, D; Genersch, E | Long-Term Temporal Trends of Nosema spp. Infection Prevalence in Northeast Germany: Continuous Spread of Nosema ceranae, an Emerging Pathogen of Honey Bees (Apis mellifera), but No General Replacement of Nosema apis | FRONTIERS IN CELLULAR AND INFECTION MICROBIOLOGY | 2017 | WOS research | No | Yes | No | - |
| Gregorc, A; Knight, PR; Adamczyk, J | Powdered sugar shake to monitor and oxalic acid treatments to control varroa mites (Varroa destructor Anderson and Trueman) in honey bee (Apis mellifera) colonies | JOURNAL OF APICULTURAL RESEARCH | 2017 | WOS research | No | Yes | No | - |
| Kevill, JL; Highfield, A; Mordecai, GJ; Martin, SJ; Schroeder, DC | ABC Assay: Method Development and Application to Quantify the Role of Three DWV Master Variants in Overwinter Colony Losses of European Honey Bees | VIRUSES-BASEL | 2017 | WOS research | No | Yes | No | - |
| Mahmood, R; Asad, S; Ahmadi, W; Sarwar, G; Rafique, MK; Islam, N; Qadir, ZA; Ul Abiden, Z | Efficacy of Screen Bottom Board Tray with and without Soft Chemicals for Controlling Varroa destructor in Honeybee Colonies | PAKISTAN JOURNAL OF ZOOLOGY | 2017 | WOS research | No | Yes | No | - |
| Requier, F; Odoux, JF; Henry, M; Bretagnolle, V | The carry-over effects of pollen shortage decrease the survival of honeybee colonies in farmlands | JOURNAL OF APPLIED ECOLOGY | 2017 | WOS research | No | Yes | No | - |
| Siede, R; Faust, L; Meixner, MD; Maus, C; Grünewald, B; Büchler, R | Performance of honey bee colonies under a long-lasting dietary exposure to sublethal concentrations of the neonicotinoid insecticide thiacloprid | PEST MANAGEMENT SCIENCE | 2017 | WOS research | No | Yes | No | - |
| Switanek, M; Crailsheim, K; Truhetz, H; Brodschneider, R | Modelling seasonal effects of temperature and precipitation on honey bee winter mortality in a temperate climate | SCIENCE OF THE TOTAL ENVIRONMENT | 2017 | WOS research | No | Yes | No | - |
| Vargas, M; Arismendi, N; Riveros, G; Zapata, N; Bruna, A; Vidal, M; Rodríguez, M; Gerding, M | Viral and intestinal diseases detected in Apis mellifera in Central and Southern Chile | CHILEAN JOURNAL OF AGRICULTURAL RESEARCH | 2017 | WOS research | No | Yes | No | - |
| Wu, YF; Dong, XF; Kadowaki, T | Characterization of the Copy Number and Variants of Deformed Wing Virus (DWV) in the Pairs of Honey Bee Pupa and Infesting Varroa destructor or Tropilaelaps mercedesae | FRONTIERS IN MICROBIOLOGY | 2017 | WOS research | No | Yes | No | - |
| Carroll, MJ; Meikle, WG; McFrederick, QS; Rothman, JA; Brown, N; Weiss, M; Ruetz, Z; Chang, E | Pre-almond supplemental forage improves colony survival and alters queen pheromone signaling in overwintering honey bee colonies | APIDOLOGIE | 2018 | WOS research | No | Yes | No | - |
| Maucourt, S; Fournier, V; Giovenazzo, P | Comparison of three methods to multiply honey bee (Apis mellifera) colonies | APIDOLOGIE | 2018 | WOS research | No | Yes | No | - |
| Molineri, A; Giacobino, A; Pacini, A; Cagnolo, NB; Merke, J; Orellano, E; Bertozzi, E; Zago, L; Aignasse, A; Pietronave, H; Rodríguez, G; Crisanti, P; Palacio, MA; Signorini, M | Environment and Varroa destructor management as determinant of colony losses in apiaries under temperate and subtropical climate | JOURNAL OF APICULTURAL RESEARCH | 2018 | WOS research | No | Yes | No | - |
| Siede, R; Meixner, MD; Almanza, MT; Schöning, R; Maus, C; Büchler, R | A long-term field study on the effects of dietary exposure of clothianidin to varroosis-weakened honey bee colonies | ECOTOXICOLOGY | 2018 | WOS research | No | Yes | No | - |
| Diao, QY; Yang, DH; Zhao, HX; Deng, S; Wang, XL; Hou, CS; Wilfert, L | Prevalence and population genetics of the emerging honey bee pathogen DWV in Chinese apiculture | SCIENTIFIC REPORTS | 2019 | WOS research | No | Yes | No | - |
| Dynes, TL; Berry, JA; Delaplane, KS; Brosi, BJ; de Roode, JC | Reduced density and visually complex apiaries reduce parasite load and promote honey production and overwintering survival in honey bees | PLOS ONE | 2019 | WOS research | No | Yes | No | - |
| Erban, T; Vaclavikova, M; Tomesova, D; Halesova, T; Hubert, J | tau-Fluvalinate and other pesticide residues in honey bees before overwintering | PEST MANAGEMENT SCIENCE | 2019 | WOS research | No | Yes | No | - |
| Flores, JM; Gil-Lebrero, S; Gámiz, V; Rodríguez, MI; Ortiz, MA; Quiles, FJ | Effect of the climate change on honey bee colonies in a temperate Mediterranean zone assessed through remote hive weight monitoring system in conjunction with exhaustive colonies assessment | SCIENCE OF THE TOTAL ENVIRONMENT | 2019 | WOS research | No | Yes | No | - |
| Goodrich, BK | Do more bees imply higher fees? Honey bee colony strength as a determinant of almond pollination fees | FOOD POLICY | 2019 | WOS research | No | Yes | No | - |
| Howes, C; Symes, CT; Byholm, P | Evidence of large-scale range shift in the distribution of a Palaearctic migrant in Africa | DIVERSITY AND DISTRIBUTIONS | 2019 | WOS research | No | Yes | No | - |
| Kiprijanovska, H; Lazarevska, S; Golubovski, M; Uzunov, A | First report on the presence of Megaselia rufipes (Meigen, 1804) and Megaselia praeacuta (Schmitz, 1919) in honey bee colonies in the Republic of Macedonia | JOURNAL OF APICULTURAL RESEARCH | 2019 | WOS research | No | Yes | No | - |
| Rucker, RR; Thurman, WN; Burgett, M | Colony Collapse and the Consequences of Bee Disease: Market Adaptation to Environmental Change | JOURNAL OF THE ASSOCIATION OF ENVIRONMENTAL AND RESOURCE ECONOMISTS | 2019 | WOS research | No | Yes | No | - |
| Smart, MD; Otto, CRV; Lundgren, JG | Nutritional status of honey bee (Apis mellifera L.) workers across an agricultural land-use gradient | SCIENTIFIC REPORTS | 2019 | WOS research | No | Yes | No | - |
| Zemeckis, R; Dautarte, A; Kretavicius, J; Drozdz, J | Effects of winter and spring rape seed treatment with neonicotinoids on honey bees | ZEMDIRBYSTE-AGRICULTURE | 2019 | WOS research | No | Yes | No | - |
| Bixby, M; Hoover, SE; McCallum, R; Ibrahim, A; Ovinge, L; Olmstead, S; Pernal, SF; Zayed, A; Foster, LJ; Guarna, MM | Honey Bee Queen Production: Canadian Costing Case Study and Profitability Analysis | JOURNAL OF ECONOMIC ENTOMOLOGY | 2020 | WOS research | No | Yes | No | - |
| Büchler, R; Uzunov, A; Kovacic, M; Presern, J; Pietropaoli, M; Hatjina, F; Pavlov, B; Charistos, L; Formato, G; Galarza, E; Gerula, D; Gregorc, A; Malagnini, V; Meixner, MD; Nedic, N; Puskadija, Z; Rivera-Gomis, J; Jenko, MR; Skerl, MIS; Vallon, J; Vojt, D; Wilde, J; Nanetti, A | Summer brood interruption as integrated management strategy for effective Varroa control in Europe | JOURNAL OF APICULTURAL RESEARCH | 2020 | WOS research | No | Yes | No | - |
| de Graaf, DC; Laget, D; De Smet, L; Boúúaert, DC; Brunain, M; Veerkamp, RF; Brascamp, EW | Heritability estimates of the novel trait 'suppressed in ovo virus infection' in honey bees (Apis mellifera) | SCIENTIFIC REPORTS | 2020 | WOS research | No | Yes | No | - |
| De la Mora, A; Emsen, B; Morfin, N; Borges, D; Eccles, L; Kelly, PG; Goodwin, PH; Guzman-Novoa, E | Selective Breeding for Low and High Varroa destructor Growth in Honey Bee (Apis mellifera) Colonies: Initial Results of Two Generations | INSECTS | 2020 | WOS research | No | Yes | No | - |
| Dittes, J; Schäfer, MO; Aupperle-Lellbach, H; Mülling, CKW; Emmerich, IU | Overt Infection with Chronic Bee Paralysis Virus (CBPV) in Two Honey Bee Colonies | VETERINARY SCIENCES | 2020 | WOS research | No | Yes | No | - |
| Dubois, E; Dardouri, M; Schurr, F; Cougoule, N; Sircoulomb, F; Thiéry, R | Outcomes of honeybee pupae inoculated with deformed wing virus genotypes A and B | APIDOLOGIE | 2020 | WOS research | No | Yes | No | - |
| Dufour, C; Fournier, V; Giovenazzo, P | The impact of lowbush blueberry (Vaccinium angustifolium Ait.) and cranberry (Vaccinium macrocarpon Ait.) pollination on honey bee (Apis mellifera L.) colony health status | PLOS ONE | 2020 | WOS research | No | Yes | No | - |
| Emsen, B; De la Mora, A; Lacey, B; Eccles, L; Kelly, PG; Medina-Flores, CA; Petukhova, T; Morfin, N; Guzman-Novoa, E | Seasonality of Nosema ceranae Infections and Their Relationship with Honey Bee Populations, Food Stores, and Survivorship in a North American Region | VETERINARY SCIENCES | 2020 | WOS research | No | Yes | No | - |
| Frizzera, D; Del Fabbro, S; Ortis, G; Zanni, V; Bortolomeazzi, R; Nazzi, F; Annoscia, D | Possible side effects of sugar supplementary nutrition on honey bee health | APIDOLOGIE | 2020 | WOS research | No | Yes | No | - |
| Galatiuk, O; Romanishina, T; Lakhman, A; Zastulka, O; Balkanska, R | Isolation and identification of Klebsiella aerogenes from bee colonies in bee dysbiosis | THAI JOURNAL OF VETERINARY MEDICINE | 2020 | WOS research | No | Yes | No | - |
| Guimaraes-Cestaro, L; Maia, TS; Martins, R; Alves, MLTMF; Otsuk, IP; Message, D; Teixeira, EW | Nosema ceranae (Microsporidia: Nosematidae) Does Not Cause Collapse of Colonies of Africanized Apis mellifera (Hymenoptera: Apidae) in Tropical Climate | SOCIOBIOLOGY | 2020 | WOS research | No | Yes | No | - |
| Lemanski, NJ; Bansal, S; Fefferman, NH | The sensitivity of a honeybee colony to worker mortality depends on season and resource availability | BMC EVOLUTIONARY BIOLOGY | 2020 | WOS research | No | Yes | No | - |
| Mahmood, R; Abu Bakar, M; Raza, MF; Qadir, ZA; Yahya, M | Efficacy of Naturally Occurring Chemicals for the Integrated Control of Varroa destructor (Anderson and Trueman) in Honeybee Colonies | PAKISTAN JOURNAL OF ZOOLOGY | 2020 | WOS research | No | Yes | No | - |
| Medici, SK; Blando, M; Sarlo, E; Maggi, M; Espinosa, JP; Ruffinengo, S; Bianchi, B; Eguaras, M; Recavarren, M | Pesticide residues used for pest control in honeybee colonies located in agroindustrial areas of Argentina | INTERNATIONAL JOURNAL OF PEST MANAGEMENT | 2020 | WOS research | No | Yes | No | - |
| Russo, RM; Liendo, MC; Landi, L; Pietronave, H; Merke, J; Fain, H; Muntaabski, I; Palacio, MA; Rodríguez, GA; Lanzavecchia, SB; Scannapieco, AC | Grooming Behavior in Naturally Varroa-Resistant Apis mellifera Colonies From North-Central Argentina | FRONTIERS IN ECOLOGY AND EVOLUTION | 2020 | WOS research | No | Yes | No | - |
| Sabahi, Q; Morfin, N; Emsen, B; Gashout, HA; Kelly, PG; Otto, S; Merrill, AR; Guzman-Novoa, E | Evaluation of Dry and Wet Formulations of Oxalic Acid, Thymol, and Oregano Oil for Varroa Mite (Acari: Varroidae) Control in Honey Bee (Hymenoptera: Apidae) Colonies | JOURNAL OF ECONOMIC ENTOMOLOGY | 2020 | WOS research | No | Yes | No | - |
| Schmolke, A; Abi-Akar, F; Roy, C; Galic, N; Hinarejos, S | Simulating Honey Bee Large-Scale Colony Feeding Studies Using the BEEHAVE Model-Part I: Model Validation | ENVIRONMENTAL TOXICOLOGY AND CHEMISTRY | 2020 | WOS research | No | Yes | No | - |
| Seeburger, VC; D'Alvise, P; Shaaban, B; Schweikert, K; Lohaus, G; Schroeder, A; Hasselmann, M | The trisaccharide melezitose impacts honey bees and their intestinal microbiota | PLOS ONE | 2020 | WOS research | No | Yes | No | - |
| Traniello, IM; Bukhari, SA; Kevill, J; Ahmed, AC; Hamilton, AR; Naeger, NL; Schroeder, DC; Robinson, GE | Meta-analysis of honey bee neurogenomic response links Deformed wing virus type A to precocious behavioral maturation | SCIENTIFIC REPORTS | 2020 | WOS research | No | Yes | No | - |
| Varikou, K; Kasiotis, KM; Bempelou, E; Manea-Karga, E; Anagnostopoulos, C; Charalampous, A; Garantonakis, N; Birouraki, A; Hatjina, F; Machera, K | A Pesticide Residues Insight on Honeybees, Bumblebees and Olive Oil after Pesticidal Applications against the Olive Fruit Fly Bactrocera oleae (Diptera: Tephritidae) | INSECTS | 2020 | WOS research | No | Yes | No | - |
| Allam, SFM; Hassan, MF; Hassan, AS; Abada, MKA | Simple approaches for environmental and mechanical management of the Varroa mite, Varroa destructor Anderson and Trueman (Parasitiformes: Varroidae), on the honey bee, Apis mellifera L. (Hymenoptera: Apidae) in Egypt | EGYPTIAN JOURNAL OF BIOLOGICAL PEST CONTROL | 2021 | WOS research | No | Yes | No | - |
| de Oliveira, VHS; Nilsson, A; Kim, H; Hallgren, G; Frössling, J; Kristiansen, LF; Forsgren, E | Honey bee pathogens and parasites in Swedish apiaries: a baseline study | JOURNAL OF APICULTURAL RESEARCH | 2021 | WOS research | No | Yes | No | - |
| Fei, CJ; Williamson, KM; Woodward, RT; McCarl, BA; Rangel, J | Honey Bees, Almonds, and Colony Mortality: An Economic Simulation of the US Pollination Market | LAND ECONOMICS | 2021 | WOS research | No | Yes | No | - |
| Ilijevic, K; Vujanovic, D; Orcic, S; Purac, J; Kojic, D; Zaric, N; Grzetic, I; Blagojevic, DP; Celic, TV | Anthropogenic influence on seasonal and spatial variation in bioelements and non-essential elements in honeybees and their hemolymph | COMPARATIVE BIOCHEMISTRY AND PHYSIOLOGY C-TOXICOLOGY & PHARMACOLOGY | 2021 | WOS research | No | Yes | No | - |
| Laomettachit, T; Liangruksa, M; Termsaithong, T; Tangthanawatsakul, A; Duangphakdee, O | A model of infection in honeybee colonies with social immunity | PLOS ONE | 2021 | WOS research | No | Yes | No | - |
| Mráz, P; Hybl, M; Kopecky, M; Bohata, A; Hosticková, I; Sipos, J; Vocadlova, K; Curn, V | Screening of Honey Bee Pathogens in the Czech Republic and Their Prevalence in Various Habitats | INSECTS | 2021 | WOS research | No | Yes | No | - |
| Ramos, F; Szawarski, N; Mitton, G; Iglesias, A; de Feudis, L; Castellini, D; Gimenez-Martínez, P; Corona, M; Eguaras, M; Maggi, M | The effect of a novel dietary supplement based on fishery industry waste hydrolysate, essential fatty acids and phytochemicals on honey bee nuclei development | JOURNAL OF APICULTURAL RESEARCH | 2021 | WOS research | No | Yes | No | - |
| Simenc, L; Knific, T; Toplak, I | The Comparison of Honeybee Viral Loads for Six Honeybee Viruses (ABPV, BQCV, CBPV, DWV, LSV3 and SBV) in Healthy and Clinically Affected Honeybees with TaqMan Quantitative Real-Time RT-PCR Assays | VIRUSES-BASEL | 2021 | WOS research | No | Yes | No | - |
| Abou-Shaara, HF | Testing the ability of chitosan nanoparticles to improve development and performance of honey bee colonies during early spring | ENTOMOLOGICAL RESEARCH | 2022 | WOS research | No | Yes | No | - |
| Alonso-Prados, E; González-Porto, AV; García-Villarubia, C; López-Pérez, JA; Valverde, S; Bernal, J; Martín-Hernández, R; Higes, M | Effects of Thiamethoxam-Dressed Oilseed Rape Seeds and Nosema ceranae on Colonies of Apis mellifera iberiensis, L. under Field Conditions of Central Spain. Is Hormesis Playing a Role? | INSECTS | 2022 | WOS research | No | Yes | No | - |
| Bahreini, R; Nasr, M; Docherty, C; de Herdt, O; Feindel, D; Muirhead, S | In Vivo Inhibitory Assessment of Potential Antifungal Agents on Nosema ceranae Proliferation in Honey Bees | PATHOGENS | 2022 | WOS research | No | Yes | No | - |
| Bodin, L; del Mazo, J; Grasl-Kraupp, B; Hogstrand, C; Leblanc, JC; Bignami, M; Hoogenboom, L; Nebbia, CS; Nielsen, E; Ntzani, E; Petersen, A; Schrenk, D; Vleminckx, C; Wallace, H; Focks, A; Gregorc, A; Metzler, M; Sgolastra, F; Tosi, S; Horvath, Z; Ippolito, A; Rortais, A; Steinkellner, H; Szentes, C; Sand, S | Evaluation of the risks for animal health related to the presence of hydroxymethylfurfural (HMF) in feed for honey bees | EFSA JOURNAL | 2022 | WOS research | No | Yes | No | - |
| Buchori, D; Rizali, A; Priawandiputra, W; Raffiudin, R; Sartiami, D; Pujiastuti, Y; Jauharlina; Pradana, MG; Meilin, A; Leatemia, JA; Sudiarta, IP; Rustam, R; Nelly, N; Lestari, P; Syahputra, E; Hasriyanti; Watung, JF; Daud, IDA; Hariani, N; Jihadi, A; Johannis, M | Beekeeping and Managed Bee Diversity in Indonesia: Perspective and Preference of Beekeepers | DIVERSITY-BASEL | 2022 | WOS research | No | Yes | No | - |
| Cormier, SB; Léger, A; Boudreau, LH; Pichaud, N | Overwintering in North American domesticated honeybees (Apis mellifera) causes mitochondrial reprogramming while enhancing cellular immunity | JOURNAL OF EXPERIMENTAL BIOLOGY | 2022 | WOS research | No | Yes | No | - |
| Cournoyer, A; Plamondon, L; Bau-Gaudreault, L; Deschamps, A; Dubreuil, P; Benoit-Biancamano, MO | Effects of Varroa destructor on Hemolymph Sugars and Secondary Infections in Honeybees (Apis mellifera) | APPLIED SCIENCES-BASEL | 2022 | WOS research | No | Yes | No | - |
| Drummond, FA | Honey Bee Exposure to the Fungicide Propiconazole in Lowbush Blueberry Fields | AGRONOMY-BASEL | 2022 | WOS research | No | Yes | No | - |
| Evans, KC; Underwood, RM; López-Uribe, MM | Combined effects of oxalic acid sublimation and brood breaks on Varroa mite (Varroa destructor) and deformed wing virus levels in newly established honey bee (Apis mellifera) colonies | JOURNAL OF APICULTURAL RESEARCH | 2022 | WOS research | No | Yes | No | - |
| Kam, JH; Brod, C; Gourde, A; Brod, M; Jeffery, G | From the Lab to the Field: Translating Applications of Near-Infrared Light from Laboratory to the Field to Improve Honeybee Mitochondrial Function and Hive Health | PHOTOBIOMODULATION PHOTOMEDICINE AND LASER SURGERY | 2022 | WOS research | No | Yes | No | - |
| Mutinelli, F; Pinto, A; Barzon, L; Toson, M | Some Considerations about Winter Colony Losses in Italy According to the Coloss Questionnaire | INSECTS | 2022 | WOS research | No | Yes | No | - |
| O'Shea-Wheller, TA; Rinkevich, FD; Danka, RG; Simone-Finstrom, M; Tokarz, PG; Healy, KB | A derived honey bee stock confers resistance to Varroa destructor and associated viral transmission | SCIENTIFIC REPORTS | 2022 | WOS research | No | Yes | No | - |
| Overturf, KA; Steinhauer, N; Molinari, R; Wilson, ME; Watt, AC; Cross, RM; vanEngelsdorp, D; Williams, GR; Rogers, SR | Winter weather predicts honey bee colony loss at the national scale | ECOLOGICAL INDICATORS | 2022 | WOS research | No | Yes | No | - |
| Reitmayer, CM; Girling, RD; Jackson, CW; Newman, TA | Repeated short-term exposure to diesel exhaust reduces honey bee colony fitness | ENVIRONMENTAL POLLUTION | 2022 | WOS research | No | Yes | No | - |
| Sagona, S; Coppola, F; Nanetti, A; Tafi, E; Palego, L; Betti, L; Giannaccini, G; Felicioli, A | Effects of Two Commercial Protein Diets on the Health of Two Imago Ages of Apis mellifera L. Reared in Laboratory | ANIMALS | 2022 | WOS research | No | Yes | No | - |
| Ziegler, C; Ueda, RM; Sinigaglia, T; Kreimeier, F; Souza, AM | Correlation of Climatic Factors with the Weight of an Apis mellifera Beehive | SUSTAINABILITY | 2022 | WOS research | No | Yes | No | - |
| Abati, R; Libardoni, G; Osowski, G; Vismara, ED; Costa-Maia, FM; Lozano, ER; Adami, PF; Potrich, M | Residual effect of imidacloprid and beta-cyfluthrin on Africanized Apis mellifera L. (Hymenoptera: Apidae) workers | APIDOLOGIE | 2023 | WOS research | No | Yes | No | - |
| Aktürk, S; Sekeroglu, A; Okuyan, S; Akdeniz, G; Arici, YK; Solmaz, S; Çakici, N; Sansar, S; Alparslan, S | The investigation of quality features of Turkish commercial honey bee queens (Apis mellifera L.) in different seasons | PAKISTAN JOURNAL OF AGRICULTURAL SCIENCES | 2023 | WOS research | No | Yes | No | - |
| Alkassab, AT; Kunz, N; Bischoff, G; Lüken, D; Janke, M; Wallner, K; Kirchner, WH; Pistorius, J | Large-scale study investigating the effects of a tank mixture containing thiacloprid-prochloraz on honey bees (Apis mellifera) | CHEMOSPHERE | 2023 | WOS research | No | Yes | No | - |
| Dawdani, S; O'Neill, M; Castillo, C; Samano, JEM; Higo, H; Ibrahim, A; Pernal, SF; Plettner, E | Effects of dialkoxybenzenes against Varroa destructor and identification of 1-allyloxy-4-propoxybenzene as a promising acaricide candidate | SCIENTIFIC REPORTS | 2023 | WOS research | No | Yes | No | - |
| García-Vicente, EJ; Martín, M; Rey-Casero, I; Pérez, A; Martínez, R; Bravo, M; Alonso, JM; Risco, D | Effect of feed supplementation with probiotics and postbiotics on strength and health status of honey bee (Apis mellifera) hives during late spring | RESEARCH IN VETERINARY SCIENCE | 2023 | WOS research | No | Yes | No | - |
| Kulhanek, K; Hopkins, BK; Sheppard, WS | Comparison of oxalic acid drip and HopGuard for pre-winter Varroa destructor control in honey bee (Apis mellifera) colonies | JOURNAL OF APICULTURAL RESEARCH | 2023 | WOS research | No | Yes | No | - |
| Kwon, M; Jung, C; Kil, EJ | Metagenomic analysis of viromes in honey bee colonies (Apis mellifera; Hymenoptera: Apidae) after mass disappearance in Korea | FRONTIERS IN CELLULAR AND INFECTION MICROBIOLOGY | 2023 | WOS research | No | Yes | No | - |
| Lee, MB | Environmental factors affecting honey bees (Apis cerana) and cabbage white butterflies (Pieris rapae) at urban farmlands | PEERJ | 2023 | WOS research | No | Yes | No | - |
| Levesque, M; Rousseau, A; Giovenazzo, P | Impacts of indoor mass storage of two densities of honey bee queens (Apis mellifera) during winter on queen survival, reproductive quality and colony performance | JOURNAL OF APICULTURAL RESEARCH | 2023 | WOS research | No | Yes | No | - |
| Medina-Flores, CA; López-Carlos, M; Carrillo-Muro, O; Gray, A | Honey Bee Colony Losses in Mexico's Semi-Arid High Plateau for the Winters 2016-2017 to 2021-2022 | INSECTS | 2023 | WOS research | No | Yes | No | - |
| Pridal, A; Musila, J; Svoboda, J | Condition and Honey Productivity of Honeybee Colonies Depending on Type of Supplemental Feed for Overwintering | ANIMALS | 2023 | WOS research | No | Yes | No | - |
| Quinlan, GM; Isaacs, R; Otto, CRV; Smart, AH; Milbrath, MO | Association of excessive precipitation and agricultural land use with honey bee colony performance | LANDSCAPE ECOLOGY | 2023 | WOS research | No | Yes | No | - |
| Rittschof, CC; Haramoto, ER; Potter, DW; Denny, AS; Harrison, JW | Impacts of fallow cropland winter weeds on honey bee pre-swarm colony growth | AGRICULTURE ECOSYSTEMS & ENVIRONMENT | 2023 | WOS research | No | Yes | No | - |
| Truong, AT; Yoo, MS; Yun, BR; Kang, JE; Noh, J; Hwang, TJ; Seo, SK; Yoon, SS; Cho, YS | Prevalence and pathogen detection of Varroa and Tropilaelaps mites in Apis mellifera (Hymenoptera, Apidae) apiaries in South Korea | JOURNAL OF APICULTURAL RESEARCH | 2023 | WOS research | No | Yes | No | - |
| Woodford, L; Sharp, G; Highet, F; Evans, DJ | All together now: Geographically coordinated miticide treatment benefits honey bee health | JOURNAL OF APPLIED ECOLOGY | 2023 | WOS research | No | Yes | No | - |
| Zhang, G; Murray, CJ; Clair, ALS; Cass, RP; Dolezal, AG; Schulte, LA; Toth, AL; O'Neal, ME | Native vegetation embedded in landscapes dominated by corn and soybean improves honey bee health and productivity | JOURNAL OF APPLIED ECOLOGY | 2023 | WOS research | No | Yes | No | - |
| BEAUCHAMP, G | EFFECTS OF ENERGY-REQUIREMENTS AND WORKER MORTALITY ON COLONY GROWTH AND FORAGING IN THE HONEY-BEE | BEHAVIORAL ECOLOGY AND SOCIOBIOLOGY | 1992 | WOS research | No | Yes | No | - |
| Gregorc, A; Planinc, I | The control of Varroa destructor in honey bee colonies using the thymol-based acaricide -: Apiguard | AMERICAN BEE JOURNAL | 2005 | WOS research | No | Yes | No | - |
| Maistrello, L; Lodesani, M; Costa, C; Leonardi, F; Marani, G; Caldon, M; Mutinelli, F; Granato, A | Screening of natural compounds for the control of nosema disease in honeybees (Apis mellifera) | APIDOLOGIE | 2008 | WOS research | No | Yes | No | - |
| vanEngelsdorp, D; Evans, JD; Saegerman, C; Mullin, C; Haubruge, E; Nguyen, BK; Frazier, M; Frazier, J; Cox-Foster, D; Chen, YP; Underwood, R; Tarpy, DR; Pettis, JS | Colony Collapse Disorder: A Descriptive Study | PLOS ONE | 2009 | WOS research | No | Yes | No | - |
| Pohorecka, K; Zdanska, D; Bober, A; Skubida, M | FIRST DETECTION OF ISRAELI ACUTE PARALYSIS VIRUS (IAPV) IN POLAND AND PHYLOGENETIC ANALYSIS OF THE ISOLATES | JOURNAL OF APICULTURAL SCIENCE | 2011 | WOS research | No | Yes | No | - |
| Pohorecka, K; Skubida, P; Miszczak, A; Semkiw, P; Sikorski, P; Zagibajlo, K; Teper, D; Koltowski, Z; Skubida, M; Zdanska, D; Bober, A | RESIDUES OF NEONICOTINOID INSECTICIDES IN BEE COLLECTED PLANT MATERIALS FROM OILSEED RAPE CROPS AND THEIR EFFECT ON BEE COLONIES | JOURNAL OF APICULTURAL SCIENCE | 2012 | WOS research | No | Yes | No | - |
| Granberg, F; Vicente-Rubiano, M; Rubio-Guerri, C; Karlsson, OE; Kukielka, D; Belák, S; Sánchez-Vizcaíno, JM | Metagenomic Detection of Viral Pathogens in Spanish Honeybees: Co- Infection by Aphid Lethal Paralysis, Israel Acute Paralysis and Lake Sinai Viruses | PLOS ONE | 2013 | WOS research | No | Yes | No | - |
| Rondeau, G; Sánchez-Bayo, F; Tennekes, HA; Decourtye, A; Ramírez-Romero, R; Desneux, N | Delayed and time-cumulative toxicity of imidacloprid in bees, ants and termites | SCIENTIFIC REPORTS | 2014 | WOS research | No | Yes | No | - |
| Giacobino, A; Rivero, R; Molineri, AI; Cagnolo, NB; Merke, J; Orellano, E; Salto, C; Signorini, M | Fumagillin control of Nosema ceranae (Microsporidia: Nosematidae) infection in honey bee (Hymenoptera: Apidae) colonies in Argentina | VETERINARIA ITALIANA | 2016 | WOS research | No | Yes | No | - |
| Szawarski, N; Quintana, S; Levy, E; Lucía, M; Abrahamovich, A; Porrini, M; Brasesco, C; Negri, P; Sarlo, G; Eguaras, M; Maggi, M | Is Acarapis woodi mite currently infesting Apis mellifera colonies in Argentina? | JOURNAL OF APICULTURAL RESEARCH | 2017 | WOS research | No | Yes | No | - |
| Zeaiter, Z; Myerscough, MR | Poor hive thermoregulation produces an Allee effect and leads to colony collapse | JOURNAL OF THEORETICAL BIOLOGY | 2020 | WOS research | No | Yes | No | - |
